# Mechanistic and structural basis of bioengineered bovine Cathelicidin-5 with optimized therapeutic activity

**DOI:** 10.1038/srep44781

**Published:** 2017-03-21

**Authors:** Bikash R. Sahoo, Kenta Maruyama, Jyotheeswara R. Edula, Takahiro Tougan, Yuxi Lin, Young-Ho Lee, Toshihiro Horii, Toshimichi Fujiwara

**Affiliations:** 1 Institute for Protein Research, Osaka University, 3-2 Yamadaoka, Suita, Osaka, 565-0871, Japan; 2 Immunology Frontier Research Center, Osaka University, 3-1 Yamadaoka, Suita, Osaka, 565-0871, Japan; 3 Research Institute for Microbial Diseases, Osaka University, 3-1 Yamadaoka, Suita, Osaka, 565-0871, Japan.

## Abstract

Peptide-drug discovery using host-defense peptides becomes promising against antibiotic-resistant pathogens and cancer cells. Here, we customized the therapeutic activity of bovine cathelicidin-5 targeting to bacteria, protozoa, and tumor cells. The membrane dependent conformational adaptability and plasticity of cathelicidin-5 is revealed by biophysical analysis and atomistic simulations over 200 μs in thymocytes, leukemia, and *E. coli* cell-membranes. Our understanding of energy-dependent cathelicidin-5 intrusion in heterogeneous membranes aided in designing novel loss/gain-of-function analogues. *In vitro* findings identified leucine-zipper to phenylalanine substitution in cathelicidin-5 (1–18) significantly enhance the antimicrobial and anticancer activity with trivial hemolytic activity. Targeted mutants of cathelicidin-5 at kink region and N-terminal truncation revealed loss-of-function. We ensured the existence of a bimodal mechanism of peptide action (membranolytic and non-membranolytic) *in vitro*. The melanoma mouse model *in vivo* study further supports the *in vitro* findings. This is the first structural report on cathelicidin-5 and our findings revealed potent therapeutic application of designed cathelicidin-5 analogues.

Antibiotic treatment of invading pathogens is one of the promising solution to manage an array of invasive microbial infections. However, the extensive use of antibiotics endorsed the natural evolution of several antibiotic or multi-drug resistant species^1^. In parallel, the alarming rise of cancer deaths remain as the principal source of investigation due to lack of suitable therapies. One major difficulty is the efficient delivery of drugs/therapeutic agents to the cancer cells through the plasma membrane^2^. Advances in the cellular uptake of drugs by polymers, micelles, liposomes, nanoparticles etc. have partially succeeded^3^. Nevertheless, the efficiency of delivery at the pathological sites is not significant due to the passive accumulation and cellular specificity. Taken together, the natural short cell-penetrating cationic peptides emerged as a central area of inquiry for their multidimensional therapeutic applications that include antimicrobial, antifungal, antiviral, antiprotozoal and antitumor activities^4^,^5^,^6^. Among these, a few antimicrobial peptides (AMP) categorized under the innate immune system are potent to tumor cells and drug-resistant microbes^7^,^8^. In general, major of the AMPs target the pathogenic cells by membranolytic mechanism, while few show non-membranolytic and intracellular activity of action^9^,^10^,^11^. Unfortunately, AMP resistant bacteria have also been evolved and the cause remains elusive. Thus, the attention has been focused on optimizing the AMP activity by modulating their cell penetrating efficacy, proteolytic stability and hemolytic activity.

From the two distinct mammalian AMP groups, i.e. defensin and cathelicidin, the latter is sequentially and structurally diverse^12^ and connected with a conserved cathelin domain (15–18 kDa). The bovine myeloid matured cathelicidin-5 or BMAP-28 is a 3.1 kDa cleaved protein that possesses antimicrobial, antiprotozoal and anticancer activity^13^,^14^,^15^ and their cytotoxic activity to human erythrocytes and neutrophils has been studied^14^,^15^. The cytotoxic activity of BMAP-28 is greater than its homologues proteins BMAP-27 and LL-37 which suggests its potential therapeutic advantages and disadvantages^14^,^16^,^17^. BMAP-28 comprised of a substantial cationic N-terminus and a hydrophobic C-terminus. The C-terminal truncation retained its antimicrobial activity while loss-of-function revealed for the hemolytic activity^16^. Thus, mutational analysis in BMAP-28 has been attempted by shrinking the hydrophobicity at the C-terminus to enhance its therapeutic activity^15^,^16^.

Although membranolytic mechanism of action by cathelicidins has been presumed, recent reports on their non-membrane lytic action extend the disagreement^11^. Hence, a profound study is needed to understand their mechanism of action which could help in designing potent analogues to act on multiple diseases. To this end, another major factor i.e. the plasma membrane heterogeneity driving BMAP-28 action need to be explored. The high toxicity of BMAP-28 minimizes its therapeutic interest and a very few studies have been carried out^14^,^15^,^18^,^19^,^20^. Undoubtedly, a significant progress has been achieved by characterizing ~2700 short peptides (http://aps.unmc.edu/AP/main.php) among which few AMPs have been successfully tested as a potential drug. However, the major problem that limits the therapeutic application is an uncontrolled toxicity. Hemolytic activity and cytotoxicity to healthy cells are of serious concern. On the contrary, optimization of natural peptides such as human cathelicidin LL-37, magainin, indolicdin etc. has been approved by the US Food and Drugs Administration (FDA) for clinical development^21^. The structural and functional characterization of human cathelicidin has been well studied in recent years^22^,^23^. Hence, a profound research in optimizing the potential cytotoxicity of BMAP-28 peptide is warranted. The selective toxicity of BMAP-28 can also be considered as an ideal target to explore the mechanism of action through molecular optimization.

To address these limitations, we explored the mechanism of BMAP-28 binding to heterogeneous membranes that mimics bacteria, leukemia, and thymocyte cells at atomistic resolution. Atomistic simulations coupled with biophysical and biochemical interpretations revealed its membrane dependent binding mechanism. The therapeutic activity of BMAP-28 and its optimized derivatives were further investigated *in vitro* and *in vivo* to ensure the cell and/or organism specific cytotoxic activity.

## Results

### Insights from atomistic simulations

**Figure 1:**
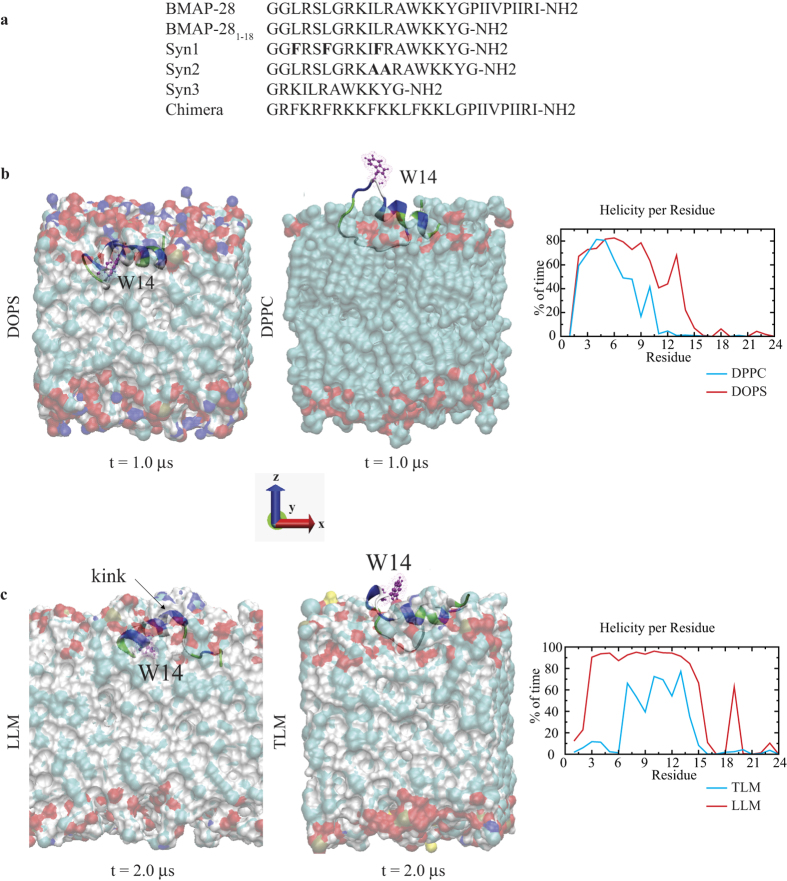
Membrane mediated conformational transition of BMAP-28. (**a**) The amino acid sequence information of BMAP-28 and its derivatives. The mutated residues are shown on bold. (**b**) The binding orientation of BMAP-28 to DOPS (left) and DPPC (center) model membranes. (**c**) Interaction of BMAP-28 with LLM (left) and TLM (center) membranes. The changes in the helical percentage of BMAP-28 with respect to simulation time are shown in the right panel of the corresponding membranes. The BMAP-28 molecule is shown as a cartoon, lipids as solid surface and the tryptophan (W14) residue as CPK in VMD. The water molecules are hidden to enhance visualization transparency.

The amino acid sequences of all synthesized peptides by reference to our structural findings in different model membrane studies are shown in Fig. 1a. The simulated BMAP-28 helical structure in aqueous solution generated an extended conformation with the commencement of β-sheet structures at each terminus and is consistent with the sequence based structure prediction (Supplementary Fig. 1a–c). In presence of bio-membranes the structural folding may vary as seen in the homologous BMAP-27 protein^24^. Our restrained molecular dynamics (MD) simulation on microsecond scale showed a helical conformation (residues 3–17) in DOPS membrane for the first 400 ns followed by the induction of a central helix kink (Supplementary Fig. 2a,b). The central kink (I10-L11) facilitate a desirable orientation for BMAP-28 penetration by stimulating an amphipathic environment at the peptide-membrane interface. In contrast, partial unfolding (residues 10–18) and aqueous phase peptide distribution was identified in DPPC (Fig. 1b, Supplementary Fig. 2b). The eukaryotic model leukemia-like membrane (LLM) system portrayed a relatively high order helix and kink formation as compared to the thymocytes-like membrane (TLM) (Fig. 1b,c). A peptide penetration of ~6 Å and relatively small ~2 Å was calculated in the anionic and zwitterionic systems, respectively (Supplementary Fig. 2c). The W14 residue was oriented towards the aqueous and membrane core regions in the zwitterionic and anionic systems, respectively (Supplementary Fig. 3a–d). At a higher peptide to lipid (P/L = 1:25) ratio, both folded and unfolded monomers in the membrane interface and water phase regions, respectively, were revealed. No stable oligomerization or membrane pore formation was observed during the 1 μs time period (Supplementary Fig. 3e). The all-atom atomistic simulation on microsecond scale could identify the peptide N-terminal association, C-terminal insertion and kink formation in the model membrane systems.

**Figure 2:**
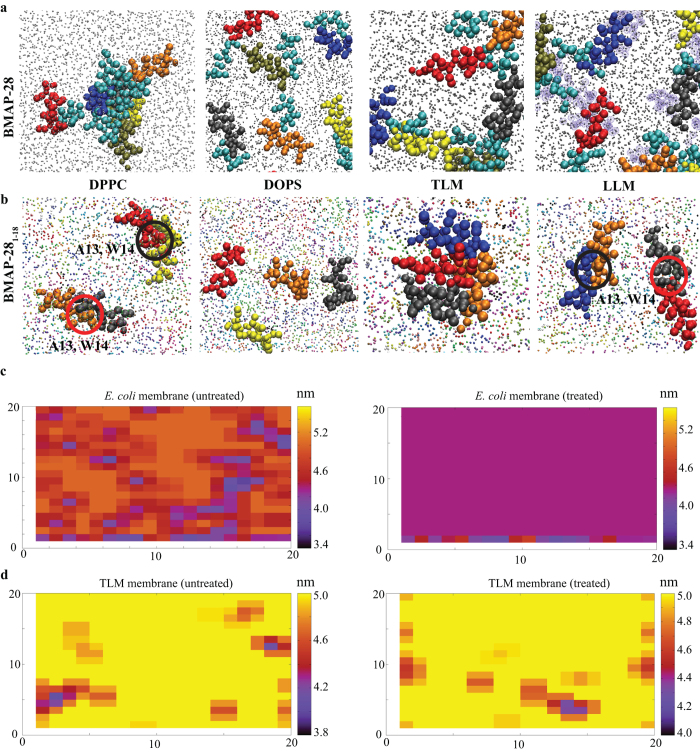
Interpretation of BMAP-28 and its derivative interaction from coarse-grained MD simulation. (**a**) The final MD snapshots showing the oligomerization of BMAP-28 in different model membrane systems. The six peptide molecules are colored differently and the C-terminal hydrophobic residues are shown in cyan. The peptide molecules are represented as VDW and the lipid molecules as CPK in VMD. The anionic lipid molecules in LLM system are shown in purple. (**b**) Membrane interaction and oligomerization of BMAP-28_1–18_. The origin of selective dimerization is shown inside the circles. The effect of the peptide on the bilayer thickness during 30 (TLM) and 100 μs (*E. coli*) calculated by GridMAT-MD tool. The effect of the peptide on the bilayer thickness in (**c**) *E. coli* (100 μs) and (**d**) TLM (30 μs) membrane systems calculated using the GridMAT-MD. The illustrations are plotted on a 20 × 20 matrix using Gnuplot. The scales at the right indicates the bilayer thickness in nanometer. The membrane without and with the peptide is denoted as untreated and treated systems, respectively.

The coarse-grained (CG) MD simulation of BMAP-28 (P/L = 1:40) showed a membrane dependent peptide binding, oligomerization and dissemination. In anionic systems, the BMAP-28 peptides were identified to interact as monomers and bordered by the anionic lipid heads (Fig. 2a). Unstable intermediate dimers/trimers were observed in the LLM system. Interestingly, acceleration of peptide self-association by C-terminal hydrophobic residues (tetramer/hexamer) was observed in the DPPC and TLM systems (Fig. 2a). The C-terminal truncated BMAP-28_1–18_ also showed a membrane dependent self-association. However, unlike the parent type, BMAP-28_1–18_ rendered a stable dimerization mediated by alanine and tryptophan residues and an unstable oligomer in the zwitterionic systems (Fig. 2b). A prominent dimeric association mode of interaction was also observed in Syn1 containing the phenylalanine zipper sequence. During the 15–30 μs, we did not observe any spontaneous pore on bilayer membrane at a concentration above the threshold reported for the homologous human cathelicidin^25^. To this end, our 100 μs MD simulation analysis of BMAP-28_1–18_ and *E. coli* membrane interaction further ensured the selective peptide distribution around the anionic (15% POPG and 5% cardiolipin) lipids. No spontaneous membrane pore formation was revealed on this time scale. But, the bilayer thickness was significantly affected as illustrated in Fig. 2c. In contrast, the effect of peptides on the membrane thickness was very limited in zwitterionic and TLM systems (Fig. 2d). This suggested in addition to P/L concentration, the peptide action is time dependent and the membrane disruption is beyond the chosen simulated time scale.

The comparative analysis also suggested the peptide aggregation and binding orientation is highly modulated by the lipid composition. The tendency of peptide oligomerization was also revealed in the aqueous solution. The ^1^H-NMR spectra of BMAP-28 and BMAP-28_1–18_ in phosphate buffer and TFE solution revealed sharp spectral peaks with small line broadening in the methyl and amide regions. No significant line broadening at the imidazole proton peak in tryptophan was observed (Supplementary Fig. 4). This suggested small oligomers (dimers/trimers) may be expected in both aqueous and hydrophobic environments; however, large oligomers are unanticipated. Taken together, we speculated the peptide aggregation in zwitterionic systems may be due to the weak membrane attachment and aqueous phase distribution. The differential penetration efficacy and self-assembly in heterogeneous membranes also indicated their distinguished cell and species specific cytotoxicity.

### Structure and energy based peptide screening

Establishing an interplay between the chemical properties of the peptide (cationicity and hydrophobicity) and the plasma membrane is crucial for toxicity modulation. To this end, we studied a chimera peptide armed with elevated amphipathicity and hydrophobicity (Fig. 1a). Interestingly, a very fast and significant loss in peptide secondary structure was observed during the 2 μs MD simulation (Supplementary Fig. 2d). This suggested a balanced cationicity and hydrophobicity is required to optimize the BMAP-28 peptide activity^26^. The identified N-terminal association, C-terminal insertion and kink regions in BMAP-28 were thus targeted with variable hydrophobicity and hydrophobic movement to create a loss-/gain-of-function peptide library (Supplementary Fig. 1c). As shown in Fig. 1a, four different BMAP-28 derivatives were designed by terminal residue truncation and kink residue mutation (Fig. 1a). To accelerate the self-association and structural stability^27^,^28^, the leucine-phenylalanine zipper derivative was designed.

**Figure 3:**
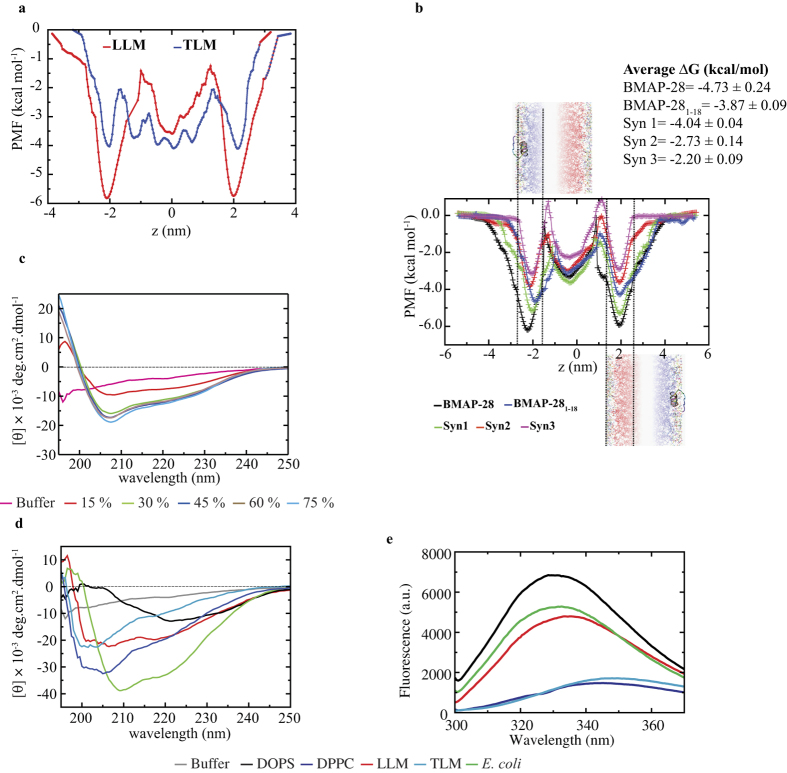
Free energy profiling of BMAP-28 using biased MD simulation. The PMF profiles of BMAP-28 in eukaryotic heterogeneous membranes. (**a**) LLM and TLM. (**b**) The membrane permeation efficacy of BMAP-28 and its derivatives in *E. coli* membrane mimicking system studied through PMF profiling. The average ΔG estimated for the outer and inner leaflet transition are shown above the PMF graphs in the cartoon format. The energy minima for different peptides are shown inside the vertically drawn dotted lines. The horizontal axis indicates the bilayer thickness where z = 0 represent the center of the lipid-bilayer. (**c**) CD spectra of BMAP-28 (0.15 mg/mL) in sodium phosphate buffer and 2,2,2-trifluoroethanol solution mixture, (**d**) CD spectra of BMAP-28 (0.15 mg/mL) in the presence of 250 µM LUV solutions. (**e**) Fluorescence spectroscopy of tryptophan (W14) in presence of LUVs at P/L = 1:40.

The free energy (ΔG) profiling in the DOPS system calculated substantial ΔG at the water/membrane interface with energy minima of −7.4 kcal mol^−1^ for BMAP-28 intrusion. The peptide transition from the water phase to lipid core revealed an oblique peptide orientation with maximum tilt angle ranging from ~45° to 60°. Transition of BMAP-28 from the water phase to membrane phase showed a negative energy minimum for all studied model membrane systems (Fig. 3). However, the ΔG minima were relatively strong in the LLM and *E. coli* systems (~−6 kcal mol^−1^) as compared to the TLM (~−2 kcal mol^−1^). The favorable ΔG in anionic systems at the water/membrane interface suggested a low energy barrier for the peptide intrusion. By contrast, the relatively weak ΔG in the TLM system suggested peptide inability to cross the rigid membrane barrier enriched in cholesterol (Fig. 3a). In *E. coli* membrane, the BMAP-28 and Syn1 peptide yielded the highest ΔG with an average value of −4.73 ± 0.24 and −4.04 ± 0.04 kcal mol^−1^, respectively; while the average ΔG for BMAP-28_1–18_ was marginally small. Remarkably, the ΔG was significantly affected for Syn2 and Syn3 peptides with an average value of −2.73 ± 0.14, and −2.20 ± 0.09 kcal mol^−1^, respectively (Fig. 3b). This suggested the onset of a potential energy barrier for the Syn2 and Syn3 peptides permeabilization into the *E. coli* membrane. Taken together, our atomistic findings correlate with the free energy results and highlights the importance of peptide kink and N-terminal residues towards the membrane intrusion and cytotoxic activity.

### Biophysical analysis of membrane binding

The CD spectrum of BMAP-28 in sodium phosphate buffer showed an extended BMAP-28 conformation; however α-helical peptide folding was ascertained with an increasing concentration of TFE solution (Fig. 3c). The CD spectrum analysis using BeStSel^29^ presented an increasing percentage of helical content from 4.7 to 38.7% and decreasing percentage of β-sheet conformation from 25.3 to 7.1% in 0 and 45% TFE solutions, respectively. The BMAP-28 derivatives also exhibited a well restrained α-helix conformation in 45% TFE solution with respect to their unfolded conformation in the aqueous buffer (Supplementary Fig. 5a). This specified the structural similarity under our selective peptide modifications. The structural folding were further analyzed by adding different concentration of 100 nm size LUVs (Table 1). Addition of anionic LUVs shifted the CD spectrum of BMAP-28 from an unfolded to folded state with negative peaks displacement from 195 nm to 208 nm (Fig. 3d). In *E. coli* LUV, a profound α-helical BMAP-28 was revealed with 38.2% helix and 7.5% β-sheet (similar to 45% TFE) conformation. Similarly, the unfolded BMAP-28 in the buffer yielded a partial folded conformation with 11.9/14.3 and 11.1/21.5% of α/β content in the heterogeneous LLM and TLM LUVs, respectively (Fig. 3d). In contrast, the CD spectra of target peptide in DPPC and TLM LUVs showed an increase percentage of random coil like conformation. The membrane mediated BMAP-28 folding that combines MD simulation and CD spectroscopy correlates one another.

The fluorescence spectroscopy of tryptophan in BMAP-28 is a suitable probe for the membrane mediated folding, penetration and self-assembly analysis. Fluorescence spectra of W14 showed a blue shift (331–335 nm) and comparatively high yield of fluorescence intensity for all anionic lipid containing LUVs (Fig. 3e). By contrast, the zwitterionic DPPC and TLM LUVs displayed relatively small quantum yield of fluorescence and the emission band at 345 and 348 nm, respectively (Fig. 3e). The blue shift and fluorescence intensity comparison ensured the polar and non-polar environment of W14 in zwitterionic and anionic LUVs, respectively. The membrane dependent distinct fluorescence emission bands proposes two major possibilities i.e. (i) The non-polar environment in anionic LUVs may be due to the substantial peptide binding and insertion, or (ii) may arise due to a relatively high order self-assembly on the membrane surface. To investigate this, the fluorescent probe 8-anilinonapthalene-1-sulfonic acid (ANS) binding assay was performed. Results showed a shift of the ANS fluorescence peak from 507 nm to 465 nm, from 493 nm to 471 nm and from 483 nm to 464 nm in DOPS, LLM and *E. coli* LUV systems, respectively (Supplementary Fig. 5b). In contrast, negligible peak shift (from 473 nm to 467 nm) and significant increase in fluorescence yield was revealed for the TLM and DPPC systems, respectively (Supplementary Fig. 5b). The ANS favorably binds to the hydrophobic residue cluster and has been reported to be accelerated in unfolded/partial folded and protein aggregation states^30^,^31^. The significant increase in the fluorescence intensity in DPPC indicated peptide aggregation or unfolding that generated a non-polar cavity for ANS binding. On the other hand, the low fluorescence yields in DOPS and *E. coli* suggested a folded or non-aggregated state of BMAP-28 binding (Supplementary Fig. 5b). The truncated BMAP-28_1–18_ also revealed similar ANS fluorescence spectra in the anionic and zwitterionic LUV systems.

**Figure 4:**
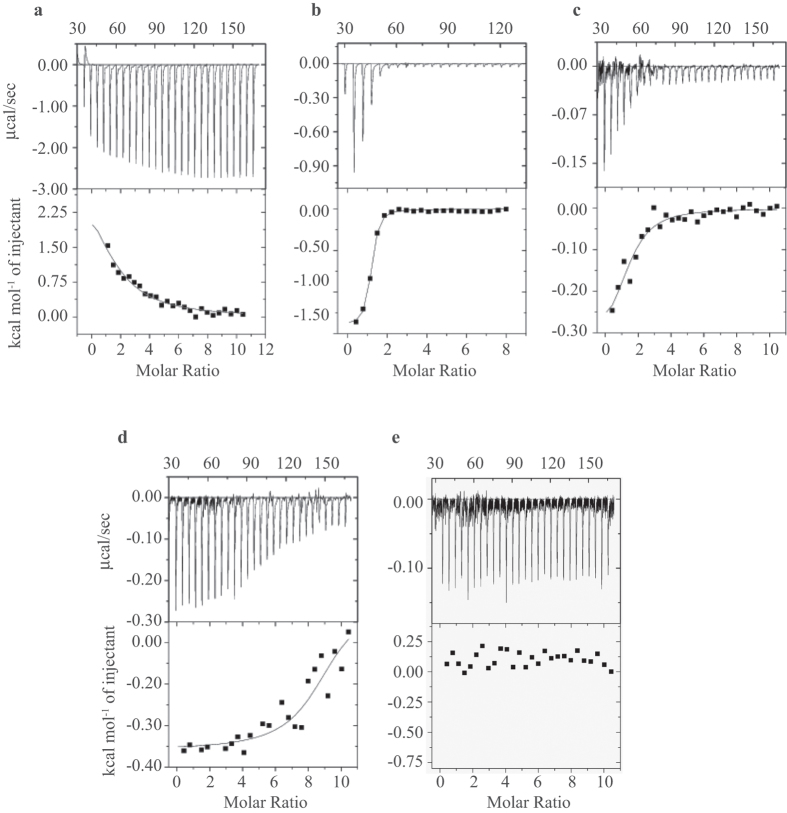
Isothermal titration calorimetry thermograms of BMAP-28 in liposomes. (**a**) DPPC; (**b**) DOPS; (**c**) *E. coli*; (d) LLM; (**e**) TLM. The lower curves show the heat of reaction measured by peak integration as a function of the P/L and the corresponding thermograms are shown above. The best fitting curve is shown in solid lines.

The thermodynamic properties of BMAP-28 binding to LUVs using ITC measurement at 37 °C were probed. An endothermic reaction was revealed for the homogeneous DPPC LUV (Fig. 4a) with a binding dissociation constant (*K*_*D*_) of 74.0 μM (Table 2). Similarly, the *K*_*D*_ for the anionic systems DOPS, LLM and *E. coli* were estimated to be 1.5 μM, 16.1 and 24.8 μM, respectively. A negative change in enthalpy in these systems indicated an exothermic reaction with small favorable enthalpic contribution (Fig. 4b–d). In contrast, the enthalpic contribution disfavors the peptide binding to the DPPC liposomes. The binding of BMAP-28 to all target LUVs except TLM was strongly driven by the entropic contribution (T*ΔS*). Interestingly, as shown in the ITC thermograms in Fig. 4e, the TLM LUVs demonstrated a non-specific binding kinetics for BMAP-28. The heat generated was comparatively low and the binding reaction did not reach saturation resulting in an uncertainty in fitting. A fast and slow saturation in anionic and zwitterionic LUVs (P/L = 1:50), respectively, can be correlated to the peptide binding efficacy and absorption. In our titration approach, the strong electrostatic binding of anionic liposomes rendered fast peptide absorption. In contrast, the low binding efficacy of BMAP-28 to TLM signified its non-specific interaction and/or weak peptide attachment/absorption. The thermodynamic parameters for each LUV systems were detailed in Table 2.

### Biological applications of target peptides

#### Antimicrobial and antimalarial efficacy

**Figure 5:**
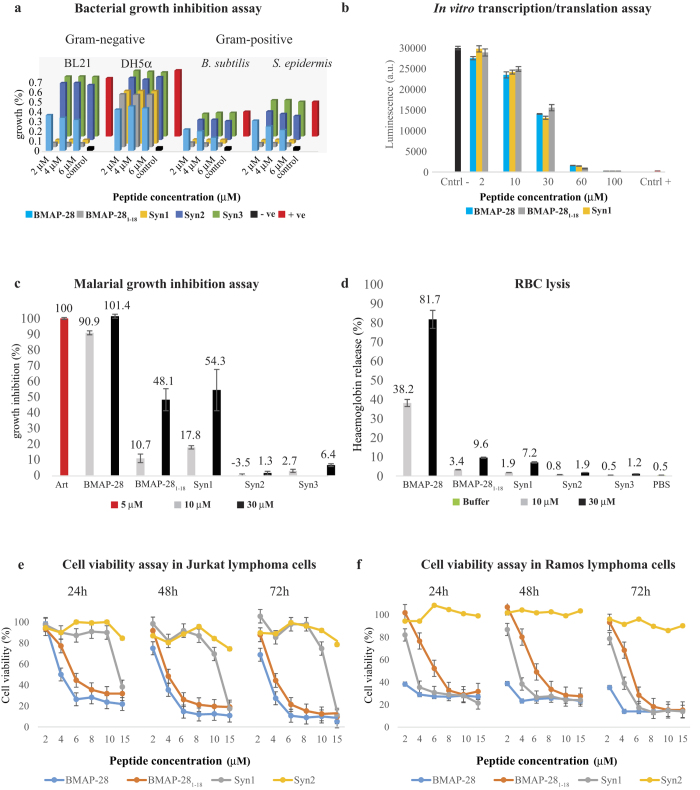
*In vitro* cytotoxicity and therapeutic activity of BMAP-28 and its derivatives. (**a**) The graphs show the antimicrobial activity at various peptide concentrations on different Gram-positive and Gram-negative bacteria. The positive and negative controls represent cells in LB medium and LB media without cells, respectively. (**b**) The intracellular binding activity of target peptides studied using the luciferase synthesis assay. The luciferase synthesis without peptide (Cntrl -) and in presence of kanamycin (Cntrl+) is used. (**c**) Effect of peptides on the percentage growth inhibition of *P. falciparum* strain. The antimalarial drug artemisinin (Art) is used as a positive control. (**d**) Hemolytic activity of target peptides is studied using the percentage release of hemoglobin. Phosphate buffer (PBS) is used as a negative control for the RBC lysis assay. Anticancer activity of BMAP-28 and its analogues in (**e**) Jurkat, and (**f**) Ramos lymphoma cells. The cell-viability assay is measured using MTT reagent after 24, 48 and 72 h incubation.

The antimicrobial assay showed low antimicrobial potency of BMAP-28 in comparison to BMAP-28_1–18_ and Syn1 with a minimum inhibition concentration (MIC) of ~2 μM for both Gram-positive and Gram-negative strains. Interestingly, the Syn2 and Syn3 peptides with kink residue mutation and N-terminal truncation, respectively, depicted a loss of activity even at one order higher concentration (Fig. 5a). The BMAP-28_1–18_ and Syn1 peptides depicted equipotent antimicrobial potency for *E. coli, Bacillus* and *Staphylococcus* strains. However, as displayed in Fig. 5a, the *E. coli* DH5α strain was shown to be resistant to all peptides even at ~5 folds above the MIC of *E. coli* BL21. The antimicrobial efficacy of wild type BMAP-28 controverts the argument of previous studies that highlighted a peptide MIC of 2.4 μM for *E. coli* DH5α strains^14^. To support our arguments, we performed different biophysical studies to provide adequate information.

**Figure 6:**
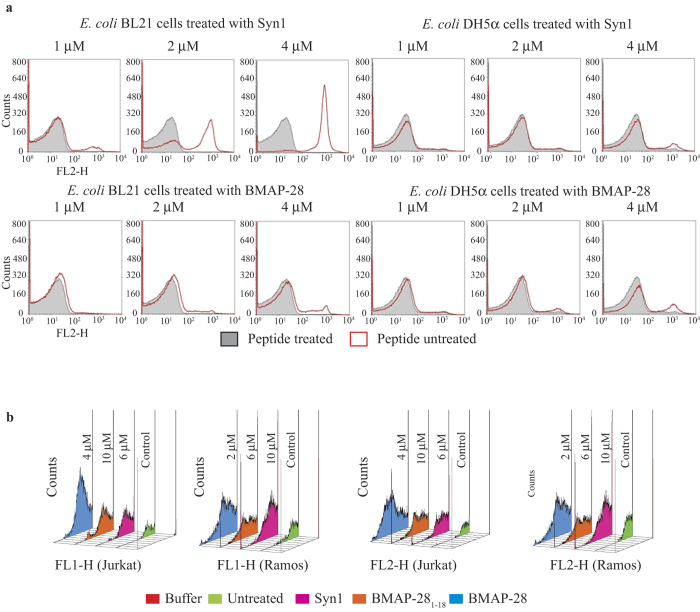
The flow cytometry analysis of the DNA intercalating PI dye in *E. coli* cells incubated at different peptide concentration. (**a**). The peptide untreated control cells are shown as gray and the change in fluorescence peaks in treated samples are presented in red. (**b**) The effect of BMAP-28 and its derivatives on lymphoma cells studied by flow cytometry. The staining of annexin V and PI in Jurkat and Ramos cells. FL1-H and FL2-H represent the fluorescence of annexin V and PI, respectively. The peptide concentrations are shown in their corresponding figure and the legends are shown at the center.

The Transmission Electron Microscopy (TEM) images showed membrane disruption and cell lysis of *E. coli* BL21 strain treated with 2 μM of Syn1 (Supplementary Fig. 6a); whereas the membrane integrity was restrained in the untreated *E. coli* BL21 cells. On the contrary, the membrane abnormalities or disruption of *E. coli* DH5α strain was not observed even at 4 μM of Syn1 (Supplementary Fig. 6b). The flow cytometry test using propidium iodide (PI) dye in Syn1 treated *E. coli* BL21 cells displayed ~100% PI influx at peptide MIC. In contrast, the *E. coli* BL21 strains showed <5% PI influx treated with 4 μM BMAP-28 (Fig. 6a; left panel). As observed in the cell assay, the *E. coli* DH5α strains depicted no significant PI influx for both peptides (Fig. 6a; right panel). This suggested the peptide resistance of *E. coli* DH5α strains can be correlated to their membrane integrity as observed by TEM analysis. Taken together, the findings suggested that the toxicity of Syn1 to *E. coli* BL21 strain was mediated by the disruption of membrane integrity. Nonetheless, alternation in the cytoplasmic concentration was also perceived in the BL21 cells. This anticipated a possible intracellular binding of the target peptide after transient membrane pore formation. Our analysis revealed the mechanism of the peptide action; however, the key molecules that mediate *E. coli* DH5α resistance remains unclear. We speculated that the resistive characteristic may accelerate through membrane surface modification and/or proteolytic enzyme secretion.

The transient cell-pore formation observed in TEM and flow cytometry pointed the possible transmission of the target α-helical peptides to the bacterial cytoplasm. The luciferase synthesis assay disclosed that the luciferase activity was reduced at 10 μM and almost disappeared at 60 μM peptide treatment (Fig. 5b). The *in vitro* transcription/translation potency of BMAP-28 and its derivatives were comparatively larger than the well-studied proline-rich^11^ antimicrobial peptides and much higher to their microbicidal MIC. Taken together, the membrane disruption ability coupled with the intracellular binding activity of the target peptides suggested that the bovine cathelicidin-5 possesses a bimodal antimicrobial mechanism of action. Antimalarial assay depicted a potential antimalarial activity of BMAP-28 (~90% parasite inhibition); whereas, the BMAP-28_1–18_ and Syn1 showed comparatively low (<20%) activity at 10 μM. The Syn2 and Syn3 analogues exhibited very low potentiality at 10 and 30 μM (Fig. 5c). The hemolysis assay for BMAP-28 presented 38% and 81% cell lysis at 10 μM and 30 μM, respectively (Fig. 5d). But, at 30 μM concentration the Syn1 and BMAP-28_1–18_ depicted ~50% parasite growth inhibition and a comparatively low hemolysis of 7.2% and 9.6%, respectively (Fig. 5d). The comparative study indicated an enhanced antimalarial activity for the designed BMAP-28 analogues.

#### Anticancer efficacy

**Figure 7:**
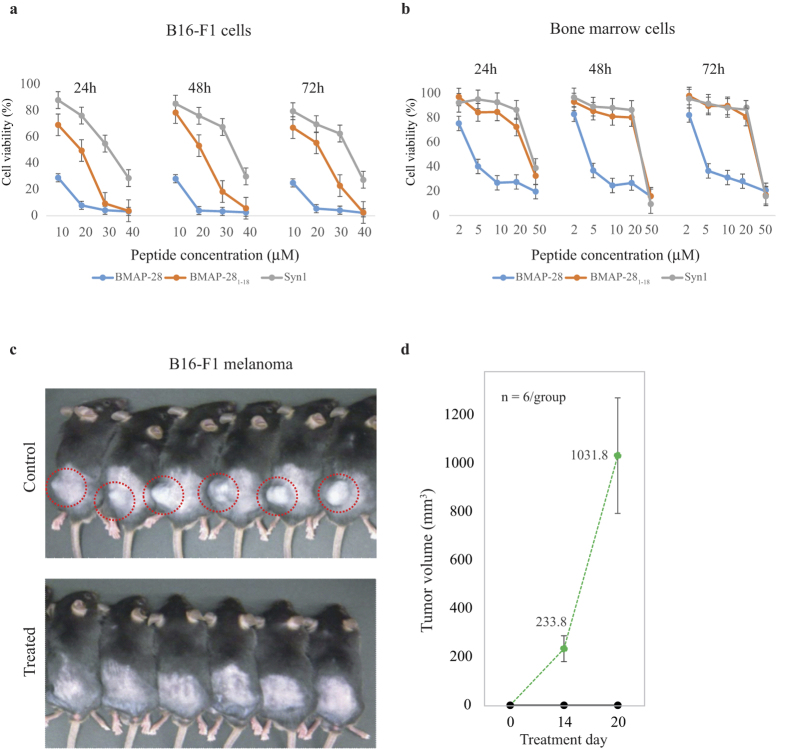
Toxic activity of BMAP-28 and its derivatives to cancer and healthy cells. Peptide activity in (**a**) mouse B16-F1 melanoma cells, and (**b**) healthy bone marrow cells. (**c**) *In vivo* images of B16-F1 bearing mice (8 weeks male) injected via subcutaneous without (upper panel) and with (lower panel) 30 μM Syn1 peptide. (**d**) Measurement of the B16-F1 tumor volume in control and Syn1 treated group (n = 6). The tumor growth is shown inside the red circles. Data were presented as mean ± s.e.m.

The BMAP-28 exhibited a significant anticancer activity at 2–4 μM against T- and B-cell lymphoma cells (Fig. 5e). The B-cell lymphoma were more susceptible to BMAP-28 (at ~2 μM; cell viability = 38%) as compared to T-cell lymphoma (Fig. 5f). Interestingly, the potent BMAP-28 analogues also possessed a substantial anticancer activity both in T- and B-cell lymphoma at a comparatively little higher concentration. The T-cell lymphoma was relatively more and less resistant to Syn1 and BMAP-28_1–18_, respectively. The Syn1 depicted a nearly similar potential anticancer activity like BMAP-28 in B-cell lymphoma (Fig. 5e,f). As conceived, the Syn2 and Syn3 peptides presented loss-of activity at a concentration higher than 30 μM. The anticancer efficacy of BMAP-28 and its derivatives against the melanoma B16-F1 cells were relatively small at 2 to 5 μM. A cell viability loss of >70% was revealed by BMAP-28 at 10 μM (Fig. 7a); whereas the BMAP-28_1–18_ and Syn1 exhibited fairly low anticancer activity at this concentration and found to be active at ~30 μM.

The BMAP-28 analogues presented low cytotoxic activity against healthy bone marrow cells at ~10 μM. In contrast, the BMAP-28 molecule was significantly cytotoxic at ~5 μM (Fig. 7b). At this concentration, a substantial loss of cell viability was ensured for macrophages, whereas no effect was seen for Syn1. The flow cytometry study ensured the binding of annexin V and PI to the lymphoma cells treated with the potential peptides at 2–10 μM as shown in Fig. 6b. This makes certain the membranolytic and peptide mediated cell proliferation activity of the target peptides. Further study of BMAP-28 analogues in RANKL-induced osteoclastogenesis showed significant activity of Syn1 at 3 μM, and a complete abolition was revealed at 10 μM (Supplementary Fig. 8). Taken together, the cell assay indicated the potential anticancer activity of Syn1 and BMAP-28_1–18_ with low hemolytic and cytotoxic activity towards the healthy cells (Figs 5 and 6). By contrast, the significant hemolytic and cytotoxic activity of BMAP-28 in healthy cells dampen its therapeutic interest.

The efficacy of target peptide Syn1 was further reveled from the *in vivo* analysis. By way of illustration, Fig. 7c shows a distinct tumor development in control and Syn1 treated mice on the day 14 and 20. The average tumor volume in control group mice was measured to be 233 and 1031 mm^3^ on day 14 and 20, respectively (Fig. 7d). In contrast, the Syn1 treated mice showed no tumor formation and were alive. The significant difference in the tumor volume correlates to the *in vitro* efficacy of Syn1 and highlights its potent anticancer activity.

## Discussion

**Figure 8:**
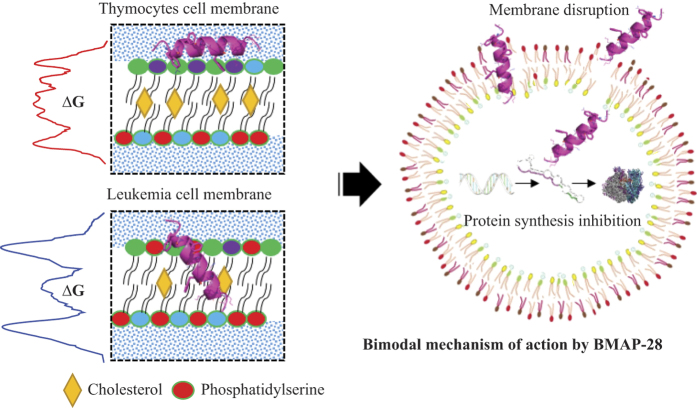
Model for BMAP-28 cell-membrane interaction and mechanism of action. The BMAP-28 peptide shows a favorable and unfavorable binding activity (left panel) in the zwitterionic (healthy cells) and anionic (cancer and microbial cells). The selective membrane binding of BMAP-28 give rise to at least two synergistic pathways of cell death (right panel).

Peptide-based rational drug designing against drug-resistant pathogens is advancing in recent years. However, the inadequate structural and functional information challenges the successful therapeutic optimization^32^. Here, we focused on modulating the inordinate cytotoxicity for a successful remedial design of the target bovine peptide whose human homologous protein is under clinical development^17^,^22^,^23^,^33^. The mechanistic and structural basis of bovine BMAP-28 activity explored here described its membrane dependent folding, kinetics and specificity through selective phospholipid binding and reorganization. Structural analysis in other AMPs using partial lipid distribution that imitates bacteria and eukaryotic cells^33^,^34^ have been amply discussed. To our knowledge, this is the first structural report that provides a comparative study of cathelicidin action on eukaryotic cancer and normal cell membrane that significantly varied on net surface charge and phospholipids distribution. The BMAP-28 binding and penetration relies on the anionic lipid distribution as reveled from our calorimetry and spectroscopy measurements. Despite of a likely membrane composition in TLM and LLM, the trivial anionic PS phospholipid variation defines the membrane attachment and binding kinetics (Fig. 8).

The atomic simulation and biophysical results ensured the lipid heterogeneity dependent BMAP-28 structural transition, reorientation and membrane intrusion. The coulombic forces coupled with the substantial hydrophobic moment reorient the BMAP-28 molecule by aligning its polar and non-polar surfaces to the membrane hydrophilic and hydrophobic surfaces, respectively. The computational findings obtained from structural and kinetic interpretation closely resemblance to the biophysical results. Thus, centering to the optimization of BMAP-28, this study proposes a synergistic combinatorial chemistry and biophysical approach for the successful therapeutic advancement. The central helix-kink identified in BMAP-28 could be crucial to provide an improved structural rearrangement on the water/membrane interface for insertion. Structural kinks also have seen in other AMPs that generates an optimized amphipathic environment for an oblique membrane permeation^35^. In addition to peptide reorientation, the CG-MD studies showed a distinguished peptide self-assembly in the vicinity of model membranes.

The membrane mediated folded and partial folded helical states of BMAP-28 correlates other class of AMPs^26^,^36^. The leucine to phenylalanine zipper substitution ascertained the differential behavior of the synthetic BMAP-28 analogue where the former facilitates membrane fusion^37^; and the latter accelerates structural integrity through dimerization^27^. The potential of mean force (PMF) profiling based screening, identified the gain/loss-of-function peptides by anticipating their ΔG barrier (Fig. 8) at the water/membrane interface. The smaller ΔG minima (Fig. 3b) and unaffected ΔG profile upon C-terminal truncation supported our assumption that the helix-kink and N-terminal regions are crucial for the peptide activity^14^,^16^. The compressive computational and experimental peptide screening procedure presented here displayed a promising approach to rationally design more efficient host-defense peptide analogues.

The monomeric binding and relatively high membrane penetration of BMAP-28 in anionic system driven by both enthalpic and entropic contributions proposed a toroidal pore mechanism of action^25^. By contrast, the peptide molecules are tightly confined to the water/membrane surface through substantial entropic influence in zwitterionic membranes. This weak binding and self-assembly could resist the peptide diffusion unless an optimum concentration is reached. As revealed from our CG-MD study, the substantial binding could obtained in the aqueous phase followed by membrane attachment. Despite of the membrane heterogeneity, these observations could be correlated to the P/L dependent cytotoxic activity of BMAP-28. At a high peptide concentration that showed hemolysis (10–30 μM), the self-assembly propensity will be much stronger in comparison to their antimicrobial and anticancer MIC. Taken together, we hypothesize that in bacteria and tumor cells, the peptide mediated lipid reorganization and substantial membrane intrusion could commence cell lysis in relatively low concentration. The proposed arguments based on peptide-membrane study could also be limited in terms of natural cell-membrane composition that contains ~50% proteins. In addition, the bio-membranes of eukaryotic and *E. coli* cells are reinforced by cytoskeletons and peptidoglycans, respectively, to provide cell resistance to environmental stress. The present study performed using artificial LUVs and lipid-bilayer lacks such consideration and is vulnerable to stress.

Although, the peptide penetration could be directly correlated to their cytotoxic action, an interconnections between the peptide folding/unfolding and active/inactive states are still confrontational. The challenge has been observed in the transient structural switching of AMPs on or within the target cell membrane. The α-helical BMAP-28 shown to be unstructured in the aqueous phase and folded upon selective target cell binding. In alliance, the folded conformation shows active cytotoxic activity at low P/L in bacteria and cancer cells *in vitro*. Thus, taken together, an assumption can be made that the bioactive conformation of BMAP-28 centers on the dynamic structural transition from extended/β-sheet to α-helix conformational state. The aim to track the time-dependent pore formation by BMAP-28 or its potential analogues was unsuccessful in the present study at 100 μs. This suggests the structural insights into a spontaneous pore formation is far beyond the selected time scale of simulation. However, the electron microscopy, flow cytometry and *in vitro* transcription/translation assay evidenced cell pore formation and protein synthesis inhibition (Fig. 8). Thus, we proposed a bimodal mechanism of antimicrobial and anticancer action for the target class of peptides (Fig. 8).

The membrane model based rational designing of potent anticancer and antimicrobial BMAP-28 analogues through combined computational and biophysical techniques supports our cell assay results. The truncated BMAP-28_1–18_ and Syn1 were more potent antimicrobial and anticancer agents which may be due to their less self-assembly propensity and high amphipathic movement. Their variable cytotoxic activity to T- and B-cells could be related to the differential membrane composition and/or enzymatic inhibition. In absence of the lipidomics information for the lymphoma, melanoma and bone marrow cells, it is difficult to correlate the peptide cytotoxicity solely based on membrane heterogeneity. However, assuming the cell-growth properties and membrane composition of the target cancer and normal cells are different, a constructive conclusion can be proposed in correlation to the biophysical and computational results. Furthermore, the membrane integrity in *E. coli* DH5α strains also ascertained existence of indirect membrane disruption pathway such as enzyme binding like penicillin, lysozyme mediated cleavage of glucosamine etc. The membrane integrity in DH5α also could be due to the host membrane surface modification and/or protease activity. These findings challenge the previous results^14^, but the cause of resistance at the molecular level remains unopened and is beyond the scope of this study.

The loss-of-function of BMAP-28 analogues (Syn2 and Syn3) showed the reliability of PMF and atomistic structural assumptions that correlates *in vitro* results. Moreover, the BMAP-28_1–18_ and Syn1 supported the proposed mechanism of peptide optimization. Although, the anticancer and antimalarial efficacy of BMAP-28 was much stronger, the relative hemolytic and cytotoxic activity to healthy cells indicates their clinical disagreement. In contrast, the BMAP-28_1–18_ and Syn1 are proposed to be clinically advantageous. The *in vivo* test highlights the therapeutic efficacy for melanoma cancer and urge further investigation in animal models of leukemia for clinical progression.

In summary, the combined computational and experimental approach open the gateway to advance the research in host-defense peptide optimization. This holistic approach can be used to rationally design potential AMPs by studying their membrane penetration potentiality and/or intracellular activity. The optimized BMAP-28 analogues displayed an enhanced antimicrobial, anticancer and antiprotozoal activity. Their low hemolytic activity and cytotoxity to healthy cells further claimed the potent therapeutic activity. The energetically favorable and unfavorable binding of BMAP-28 suggests its cell/species specificity and is proposed to be relying on the membrane heterogeneity. In summation, the intracellular binding affinity of our target peptides that inhibits the target cell transcription/translation process propose a bimodal antimicrobial/anticancer action as illustrated in Fig. 8 (right panel). The membrane disruption and intracellular activities has also been seen in a well-studied α-helical peptide MSI-78^38^,^39^,^40^. On the whole, the projected approach shade lights on the mechanistic and structural basis of bovine cathelicidin-5 interaction at the atomic level to develop therapies against multiple diseases.

## Materials and Methods

### Materials

All the studied peptides (Fig. 1a) were synthesized and purified (>97% purity) by Scrum Inc., Japan and used without further purification. The phospholipids that includes 1,2-dihexadecanoyl-sn-glycero-3-phosphocholine (DPPC), 1-palmitoyl-2-oleoyl-sn-glycero-3-phosphoethanolamine (POPE), 1-palmitoyl-2-oleoyl-sn-glycero-3-phosphocholine (POPC), 1-Palmitoyl-2-oleoyl-sn-glycero-3-phosphoglycerol (POPG), 1, 2-Dioleoyl-sn-glycero-3-phospho-L-serine (DOPS), 1-stearoyl-2-hydroxy-sn-glycero-3-phosphocholine (Lyso-PC), 1-palmitoyl-2-hydroxy-sn-glycero-3-phosphoethanolamine (Lyso-PE), Cardiolipin, egg sphingomyelin (SM), and cholesterol were purchased from Wako Pure Chemical Industries, Ltd. (Japan). Unless specified, the biophysical studies were conducted using 25 mM sodium phosphate buffer (pH = 6.4) solution and 100 mM NaCl at 37 °C. The peptide concentrations were measured at 280 nm using U-3000 spectrophotometer (Hitachi, Japan).

### Molecular mechanics and molecular dynamics simulation

The three dimensional structure of BMAP-28 was built using ab-initio modeling method^41^ in reference to the circular dichroism, helical wheel and secondary structure information. A peptide chimera comprised of high cationic N-terminus of BMAP-27 (PBD ID: 2KET) and strong hydrophobic (19–27) C-terminus was prepared. Unbiased and biased all-atom and coarse-grained (CG) MD simulations were performed on GROMACS 5.0.1^42^ using amber99sb-ildn^43^, charmm36^44^ and Martini^45^ force fields as described in our previous report^24^. The MD simulations were performed in homogeneous/heterogeneous lipid-bilayer systems as listed in Supplementary Table 1. The lipid composition of heterogeneous membrane systems i.e. thymocytes-like (TLM), leukemia-like (LLM) and *E. coli* like membranes were directly referred from the experimental molar concentration as listed in Table 1^46^,^47^. A total of 100 windows with different peptide initial position was selected and each window was simulated for 100 ns to enable adequate sampling configuration.

### Antimicrobial assay

The *Escherichia coli* strains BL21 and DH5α were obtained from Novagen and Takara Clontech, respectively. The *Bacillus subtilis* (3009) and *Staphylococcus epidermis* (12993) were purchased from NITE Biological Resource Center (NBRC, Japan). The bacterial growth procedures followed in this study were described elsewhere^38^. The mid-log phase microbial growth (~4 × 10^7^ cells/mL, 5 μL) was used for growth assay in a 96-well plate (5 replicates) at a variable peptide concentration incubated at 37 °C for 12–48 h.

### Malarial and hemolysis assay

*Plasmodium falciparum* strain 3D7 was cultured in RPMI-1640 supplemented with 25 mg/l L-Glutamine; HEPES, NaHCO_3_; 50 mg/l Hypoxanthin; Gentamicien; 10% human serum; and RBCs at 3% hematocrit in an atmosphere of 5% CO_2_, 5% O_2_, and 90% N_2_ at 37°C as previously described^48^. The *Plasmodium* lactate dehydrogenase (pLDH) assay was performed to measure the parasitic growth inhibition as described elsewhere^49^ with BMAP-28 and its analogues treatment. In brief, malstat reagent was added to a 96-well plate (100 μL in each well). A *Plasmodium* infected RBC mixture of 20 μL in each well was mixed thoroughly using a plate shaker. And then 10 μL of each 1 mg/ml diaphorase and Nitroblue tetrozolium (NBT) were added to each well. The solution in the 96-well plate was mixed properly until a brown color appears and the absorbance was measured at 650 nm under a plate reader (PowerWaveHT, BioTek Instruments, USA). The hemolytic assay was carried out as described earlier^50^. Briefly, RBCs prepared from fresh blood (Hematocrit ~5%) were incubated for 1 h at 37 °C after addition of test peptides. Relative hemoglobin concentration in supernatants after centrifugation at 2000× g for 5 min was monitored by measuring the absorbance at 540 nm by the plate reader (PowerWaveHT, BioTek Instruments, USA). As positive control, 100% hemolysis was taken from samples in which 2% Triton X-100 was added.

### Cell-culture and MTT assay

The T-cell lymphoma (Jurkat) and B-cell lymphoma (Ramos) were cultured using RPMI-1640 medium supplemented with 10% fetal calf serum (FCS), 100 U ml^−1^ penicillin and 100 μg ml^−1^ streptomycin. An initial cell densities of 5 × 10^3^ cells/well was uniformly maintained in a 96 well plate. The B16-F1 melanoma cells were grown in DMEM medium containing 10% FCS, 100 U ml^−1^ penicillin and 100 μg ml^−1^ streptomycin and 55 μM 2-mercaptoethanol. The cells were trypsinized and plated in a 96-well plate at a density of 5 × 10^3^ cells/well. The cells were grown overnight at 37 °C using a 5% CO_2_ incubator. The cells were treated with different peptide concentration followed by cell proliferation assay. The MTT assay kit (Cayman chemicals, USA) was used to measure the cell viability at a peptide concentration varying from 2 to 15 μM (in triplicate). Fresh culture medium and cells in medium without peptide were considered as negative and positive controls for viability assay and was monitored at 24, 48 and 72 h. The Cayman MTT cell proliferation assay protocol was followed, and the absorbance were recorded at 570 nm using a microplate reader (PowerWaveHT, BioTek Instruments, USA).

### Mice and *in vitro* osteoclast culture

The C57BL/6 mice (8 weeks old male) were generated and maintained in a pathogen-free environment as described elsewhere^51^. All animal experiments were carried out under approval from the Animal Research Committee of the Research Institute for Microbial Diseases (Osaka University, Osaka, Japan). All experiments were performed in accordance with relevant guidelines and regulations of Osaka University, Japan. The stromal cell-free osteoclast culture was performed using M-CSF-derived macrophage (MDMs) precursors. The bone marrow cells were flushed from femurs or tibias and cultured for 6 h in α-MEM supplemented with 10% FCS. The non-adherent cells were collected and plated in a 96-well plate at a density of 2 × 10^3^ cells/well with 25 ng/ml M-CSF (PeproTech, Rocky Hill, NJ). The cell cultures were rinsed with PBS after 3 days incubation, and the adherent cells were used as MDMs. The MDMs were induced to differentiate into osteoclasts in the presence of 50 ng/ml RANKL plus M-CSF. The cells were treated with different peptide concentration to investigate the effect of peptides on the osteoclastogenesis process. After 4 days, TRAP staining was performed and TRAP positive multinucleated cell numbers (>3 nuclei) were counted.

### Liposome preparation

Large Unilamellar Vesicles (LUVs) were prepared by adding appropriate molar concentration of different phospholipids (Table 1) mimicking anionic, zwitterionic, TLM, LLM, and *E. coli* cell plasma membrane^46^,^47^. The detailed procedure of liposome preparation was described elsewhere^52^. In brief, phosphate buffer assay was performed to calculate the stock concentration of different lipids suspended in chloroform. Lipid films were generated under a stream of nitrogen gas followed by drying under vacuum for 1 h. The lipid films were rehydrated using sodium phosphate buffer, and were disrupted by several freeze-thaw cycle followed by 10 times extrusions through 100 nm polycarbonate membrane. The integrity of extruded LUVs and effect of peptide on LUV size was studied by dynamic light scattering (DLS) method (Malvern Zetasizer Nano; model: ZMV2000).

### Fluorescence spectroscopy

The fluorescence spectra of BMAP-28 and its derivative in different LUV systems were obtained using an F-2500 (Hitachi, Japan) spectrometer with emission and excitation slits each set at 2.5 nm. The tryptophan residue was excited at 290 nm and emission spectra was scanned between 300 and 370 nm at 37 °C. A peptide concentration of 2.5 μM and LUV concentration was increased of 100 μM was used to analyze the membrane dependent peptide orientation and binding. The membrane mediated self-assembling potentiality of both peptides was studied by employing ANS binding assays. A final concentration of 2 μM ANS was added to different LUV systems (100 μM) at 2.5 μM peptide concentration. The fluorescence spectra were assessed at 350 nm excitation and emission wavelengths ranging from 430 to 600 nm.

### Circular dichroism (CD)

The secondary structure analysis of wild and mutated synthesized peptides in different 2,2,2-trifluoroethanol (TFE) and LUV concentrations was conducted by CD on a JASCO CD-J-820 spectropolarimeter with a path length of 0.1 cm. Constant peptide solution (0.15 mg/ml) in sodium phosphate buffer with an increasing TFE concentration (range from 15 to 75%) was used to analyze the BMAP-28 and its derivatives folding properties and effect of mutation and terminal truncation. Thereafter, the BMAP-28 folding was also studied with and without LUVs (250 μM) at 50 μM peptide concentration.

### Isothermal titration calorimetry (ITC)

Binding affinities of peptide for LUV in sodium phosphate buffer were measured through ITC experiments at 37 °C on a VP-ITC instrument (GE Healthcare). A peptide at a concentration of 50 μM in the cell was titrated with liposome solution at a concentration of 2.5 mM in the syringe. Experimental procedures included a reference power of 10 μcal/s, a filter period of 2 s, stirring speed of 307 rpm, initial delay of 1800 s, 28 injections of volume 10 μL each, and time spacing of 300 s. ITC data peak integration with automated baseline adjustment was performed using Microcal program.

### Flow cytometry test

The effect of peptides on the cell-membrane integrity of *E. coli* BL21 and DH5α and lymphoma cells was studied using the flow cytometer analysis as described elsewhere^53^. The cells were cultured and washed three times using PBS buffer and incubated for 30 minutes with peptides at different concentration. The incubated lymphoma cells were further processed referring to the annexin V-FITC apoptosis kit. Peptide treated and untreated cells (*E. coli*/lymphoma) were stained with 10 μg ml^−1^ propidium iodide (PI) and incubated for additional 30 min. The incubated cells were washed using PBS to remove unbound dye and flow cytometry analysis was carried out using the FACSCalibur and analyzed by CellQuest Pro Version 5.2.1 software (BD Biosciences, NJ, USA).

### Transmission Electron Microscopy (TEM)

*E. coli* DH5α and BL21 strains were subjected to TEM analysis. *E. coli* strains were cultured at 37°C in LB broth to mid-log phase and harvested by centrifugation at 5000 g for 5 minutes. The cell pellets were re-suspended in fresh LB media with an OD_600_ = 0.18. A peptide (Syn1) solution at a concentration of 2 μM (MIC) was added to *E. coli* DH5α and BL21 strains and the samples were incubated at 37°C for 1 hour. The culture was centrifuged at 5000 g for 5 minutes and the cell pellets were collected. The cell pellets were washed twice and re-suspended in 1 mL fixation buffer that contains 2% formaldehyde, 2.5% glutaryldehide in 0.1 M phosphate buffer (pH = 7.4). The fixed bacterial cells were observed under the microscope, JEM-1011 (JEOL, Tokyo, Japan).

### Luciferase synthesis assay

Luciferase synthesis from Luciferase T7 Control DNA plasmid (L482B, Promega) was examined using *E. coli* T7 S30 Extract System (L1130, Promega). The complete protocol for reaction mixture and final solution preparation was followed as described elsewhere^38^. Peptide concentration of 2, 10, 30, 60 and 100 μM were used for our analysis. Kanamycin (500 μg/mL) and RNase free distilled water was taken as a positive and negative control, respectively. Luciferase Assay Reagent (E1500, Promega) was used to monitor the luciferase luminescence and the samples were measured using a spectrophotometer (Powerscan HT, Biotek Instruments, USA).

### *In vivo*

Healthy C57BL/6 mice (n = 6) were administered with 30 μM Syn1 peptide mixed with 1 × 10^6^ B16-F1 melanoma cells in 0.1 ml of saline via subcutaneous injections. As a control, six mice were implanted with 1 × 10^6^ B16-F1 melanoma cells in 0.1 ml of saline. The growth size of tumors were monitored in every two days and the tumor volume was measured by a dial-caliper on day 14 and 20 using the formula volume = width^2^ × length × 0.52.

## Additional Information

**How to cite this article:** Sahoo, B. R. *et al*. Mechanistic and structural basis of bioengineered bovine Cathelicidin-5 with optimized therapeutic activity. *Sci. Rep.* **7**, 44781; doi: 10.1038/srep44781 (2017).

**Publisher's note:** Springer Nature remains neutral with regard to jurisdictional claims in published maps and institutional affiliations.

## Supplementary Material

Supplementary Information

## Figures and Tables

**Table 1:** Lipid composition in different membrane systems.

*In vitro* (Large Unilamellar Vesicles)			
Lipids	Thymocytes-like (mol %)^47^	Leukemia-like (mol %)^47^	*E. coli* like (mol %)^46^
Phosphatidylcholine	30.0	38.0	0.0
Phosphatidylethanolamine	11.0	22.0	80.0
Phosphatidylserine	**0.0**	7.0	0.0
Cholesterol	42.5	24.0	0.0
Sphingomyelin	**10.0**	0.0	0.0
*Lyso*-phosphatidylcholine	2.0	2.0	0.0
*Lyso*-phosphatidylethnolamine	1.5	2.0	0.0
Phosphatidylglycerol	0.0	0.0	15.0
Phosphatidylinsitol	3.0	5.0	0.0
Cardiolipin	0.0	0.0	5.0
***In silico*** (**Phospholipid**-**bilayer**)			
Phosphatidylcholine	30.0	38.0	0.0
Phosphatidylethanolamine	11.0	22.0	80.0
Phosphatidylserine	5.5	7.0	0.0
Cholesterol	42.5	24.0	0.0
Sphingomyelin	4.5	0.0	0.0
*Lyso*-phosphatidylcholine	2.0	2.0	0.0
*Lyso*-phosphatidylethnolamine	1.5	2.0	0.0
Phosphatidylglycerol	0.0	0.0	15.0
Phosphatidylinsitol	3.0	5.0	0.0
Cardiolipin	0.0	0.0	5.0

The molar concentration shown in bold are different from the experimental lipid compositions. The percentage of anionic lipids in thymocytes-like membrane has been reciprocated with sphingomyelin.

**Table 2:** Thermodynamic parameters obtained by isothermal titration calorimetry for BMAP-28 binding to LUVs.

LUV	*ΔG* (kcal mol^−1^)	*ΔH* (kcal mol^−1^)	*ΔS* (cal mol^−1^ k^−1^)	T*ΔS* (kcal mol^−1^)	K_association_ (mol^−1^)
DPPC	−5.88	3.73	31.0	9.61	1.35 × 10^4^
DOPS	−8.25	−1.40	22.1	6.85	6.52 × 10^5^
TLM	—	—	—	—	—
LLM	−6.78	−0.35	20.8	6.48	6.19 × 10^4^
*E. coli*	−6.54	−0.34	20.0	6.20	4.02 × 10^4^
