# Supplementary material for: Mechanistic and structural basis of bioengineered bovine Cathelicidin-5 with optimized therapeutic activity
Source: Sci Rep. 2017 Mar 21;7:44781. doi: 10.1038/srep44781 (PMC5359555; doi:10.1038/srep44781)
Supplement: Supplementary Information [file srep44781-s1.pdf]

## Supplementary information:

# Mechanistic and structural basis of bioengineered bovine Cathelicidin-5 with optimized therapeutic activity

Bikash R Sahoo [1], Kenta Maruyama [2,†], Jyotheeswara R Edula [3,†], Takahiro Tougan [3,†], Yuxi Lin [1,†], Young-Ho Lee [1, †], Toshihiro Horii [3], Toshimichi Fujiwara [1,*]

[1] Institute for Protein Research, Osaka University

[2] Immunology Frontier Research Center, Osaka University

[3] Research Institute for Microbial Diseases, Osaka University

[*] **Correspondence**

E-mail: tfjwr@protein.osaka-u.ac.jp

Institute for Protein Research, Osaka University, 3-2 Yamadaoka, Suita, Osaka 565-0871, Japan

[†] These authors contributed equally

## Supplementary results:

### Effect of peptides on size distribution of LUVs

The Z-average size of all LUVs after polycarbonate membrane extrusion was in the range of ~ 100-125 nm as revealed in DLS analysis and showed a monomodal size distribution. The peptide treated (P/L = 1:100) LUVs presented insignificant changes in the average sizes. At this concentration, the treated DOPS LUVs showed a size alteration of ~ 10 nm with monomodal size distribution. The peptide effect on DPPC-LUV was relatively (~ 20 nm) high; however no polydispersity was observed. A very small size alteration (~ 4 nm) was revealed in the TLM-LUV system with monodisperse size distribution and suggested a weak peptide activity on LUV membrane disintegration (Figure S7). The restrain homogeneity of LUV and small deviation in size with BMAP-28 peptide treatment indicated the membrane integrity and non-micellar formation. In LLM system, a heterogeneous size distribution was revealed; however no significant changes were perceived (Figure S7). From the size distribution analysis, we speculated a non-membranolytic action of BMAP-28 at moderate P/L concentration. Interestingly, at a higher P:L (1:20), we observed significant changes in the size distribution of DOPS LUVs. Bimodal distribution of DOPS LUVs was revealed for the BMAP-28 treated systems; on the other hand, monomodal size distribution was identified in Syn1 and BMAP-28$_{1-18}$ treated systems. However, the changes in the average hydrodynamic diameter of peptide treated LUVs were significant for the BMAP-28 and BMAP-28$_{1-18}$ peptides.

**Supplementary Table 1.** Detail parameters of the simulated systems.

| MD systems | Force field | P:L | Solvent | Time (μs) |
|---|---|---|---|---|
| **All-atom MD of BMAP-28** | | | | |
| Aqueous | amber99sb-ildn | N/A | 3863 | 1 |
| DOPS | amber99sb-ildn | 1:64 | 3278 | 1 |
| DPPC | amber99sb-ildn | 1:64 | 2806 | 1 |
| TLM | charmm36 | 1:64 | 3643 | 2 |
| LLM | charmm36 | 1:64 | 3615 | 2 |
| LLM | charmm36 | 1:25 | 7701 | 1 |
| Chimera | charmm36 | 1:64 | 3602 | 2 |
| **Coarse-grain MD** | | | | |
| DPPC | martini | 1:40 | 5072 | 15 |
| DOPS | martini | 1:40 | 4776 | 15 |
| LLM | martini | 1:40 | 4986 | 30 |
| TLM | martini | 1:40 | 5073 | 30 |
| *E. coli* like | martini | 1:25 | 3195 | 100 |
| **Umbrella sampling systems** | | | | |
| **Simulation type** | **Force field** | **P:L** | **Time × sampling size** | |
| LLM/TLM systems | martini | 1:100 | 100 ns × 100 | |
| *E. coli* systems | martini | 1:100 | 100 ns × 100 | |

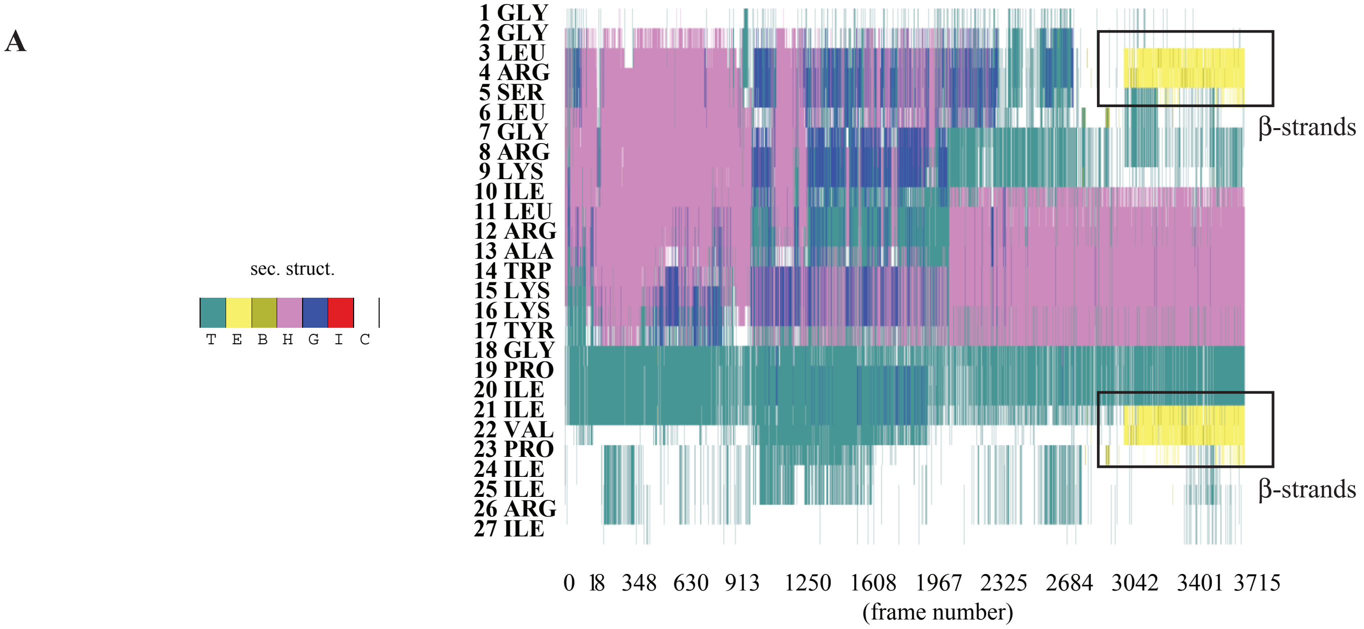


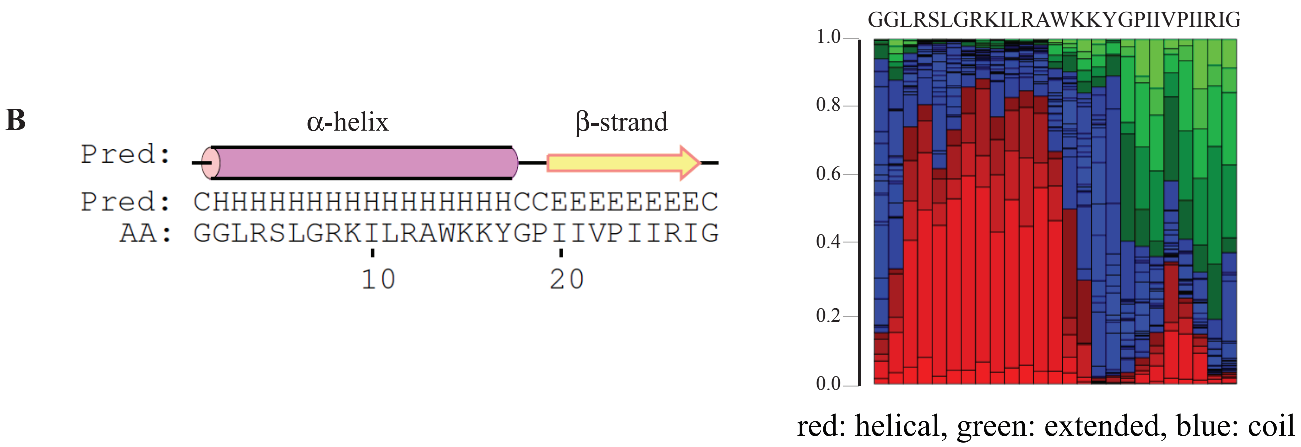


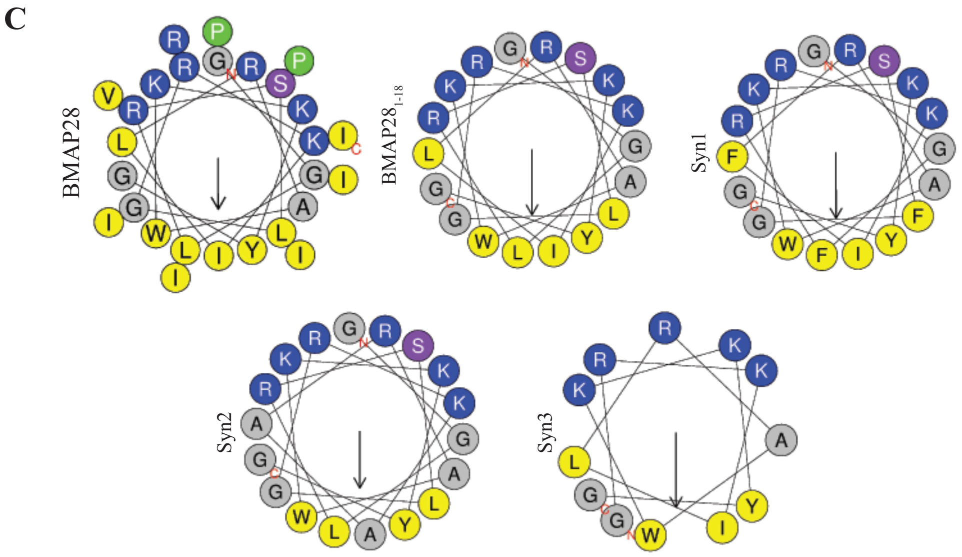


| Peptide | H | μH |
|---|---|---|
| BMAP-28 | 0.55 | 0.52 |
| BMAP28$_{1-18}$ | 0.24 | 0.62 |
| Syn1 | 0.26 | 0.63 |
| Syn2 | 0.08 | 0.55 |
| Syn3 | 0.17 | 0.70 |

Fig. S1. Analysis of secondary structure of BMAP-28. (A) The secondary structure transitions of BMAP-28 in aqueous solution during 1 μs time period are plotted against the MD trajectory. The legend of secondary structural units is shown on the left side (t=turn; E=β-sheet; B=isolated bridge; H=α-helix; G=3$_{10}$-helix; I= Pi-helix; C=coil). (B) Secondary structure prediction from BMAP-28 amino acid sequence in PSIPRED (left) and PEP-FOLD (right) program.
(C) Helical projection of BMAP-28 and its derivatives. The hydrophobicity and hydrophobic moment are denoted as "H" and "μH", respectively.

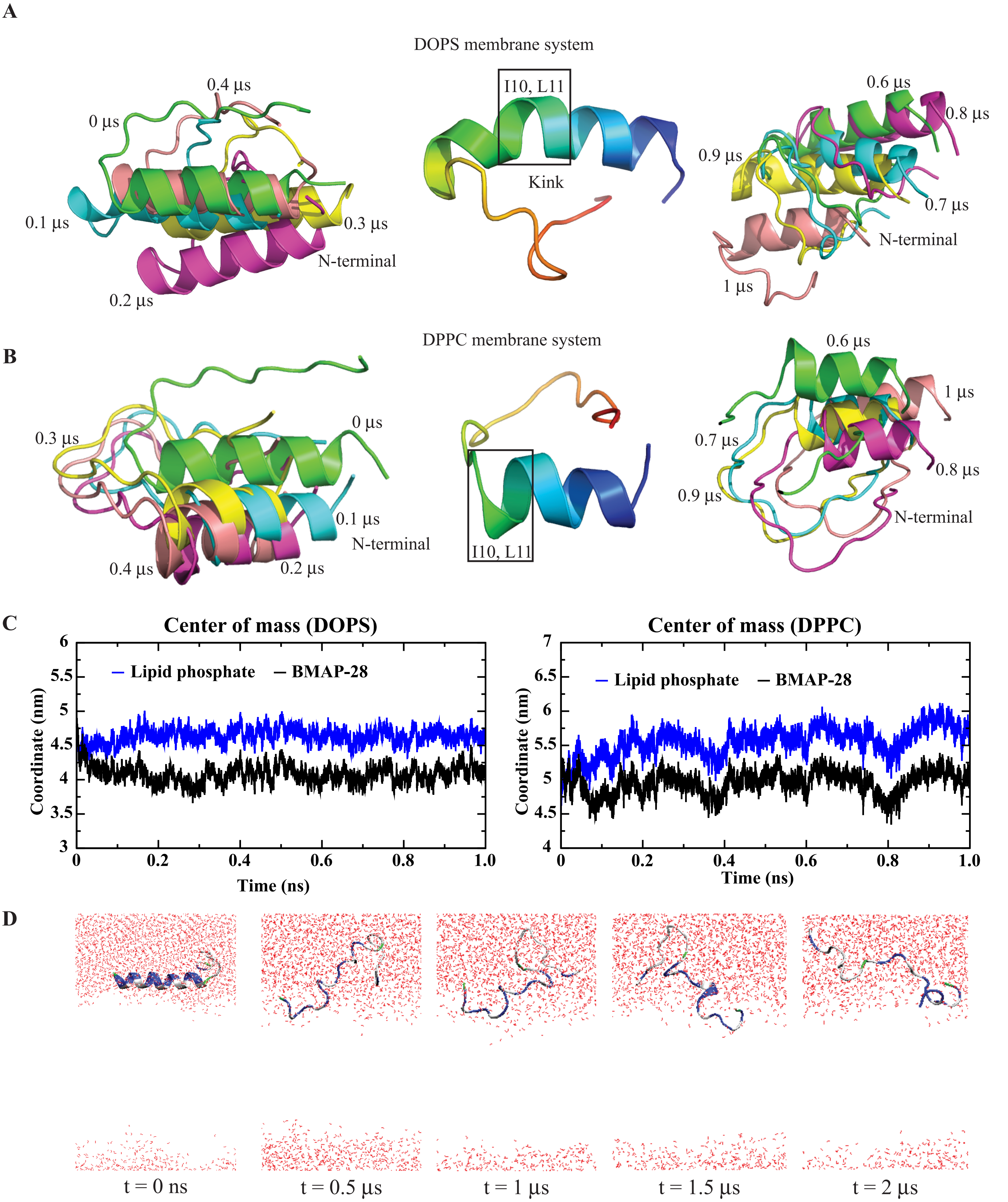


Fig. S2. MD snapshots of BMAP-28 interacting with (A) anionic and (B) zwitterionic membrane systems during 1 μs time period. The peptide molecule is shown in cartoon and the induced kink is shown inside the black boxes. The membrane dependent structural folding/unfolding of BMAP-28 is displayed in every 100 ns time period and are superimposed using PyMOL. (C) Measurement of membrane penetration by BMAP-28 through their relative center of mass (COM) alignment. The COM is calculated for all lipid phosphate atoms present in the exofacial membrane surface and for the backbone atoms of BMAP-28. (D) Snapshots showing the structural changes in the chimera peptide during 2 μs all-atom MD simulation in VMD. Protein and water molecules are represented by cartoon and line, respectively. The lipid bilayer at center is hidden to optimize the illustration.

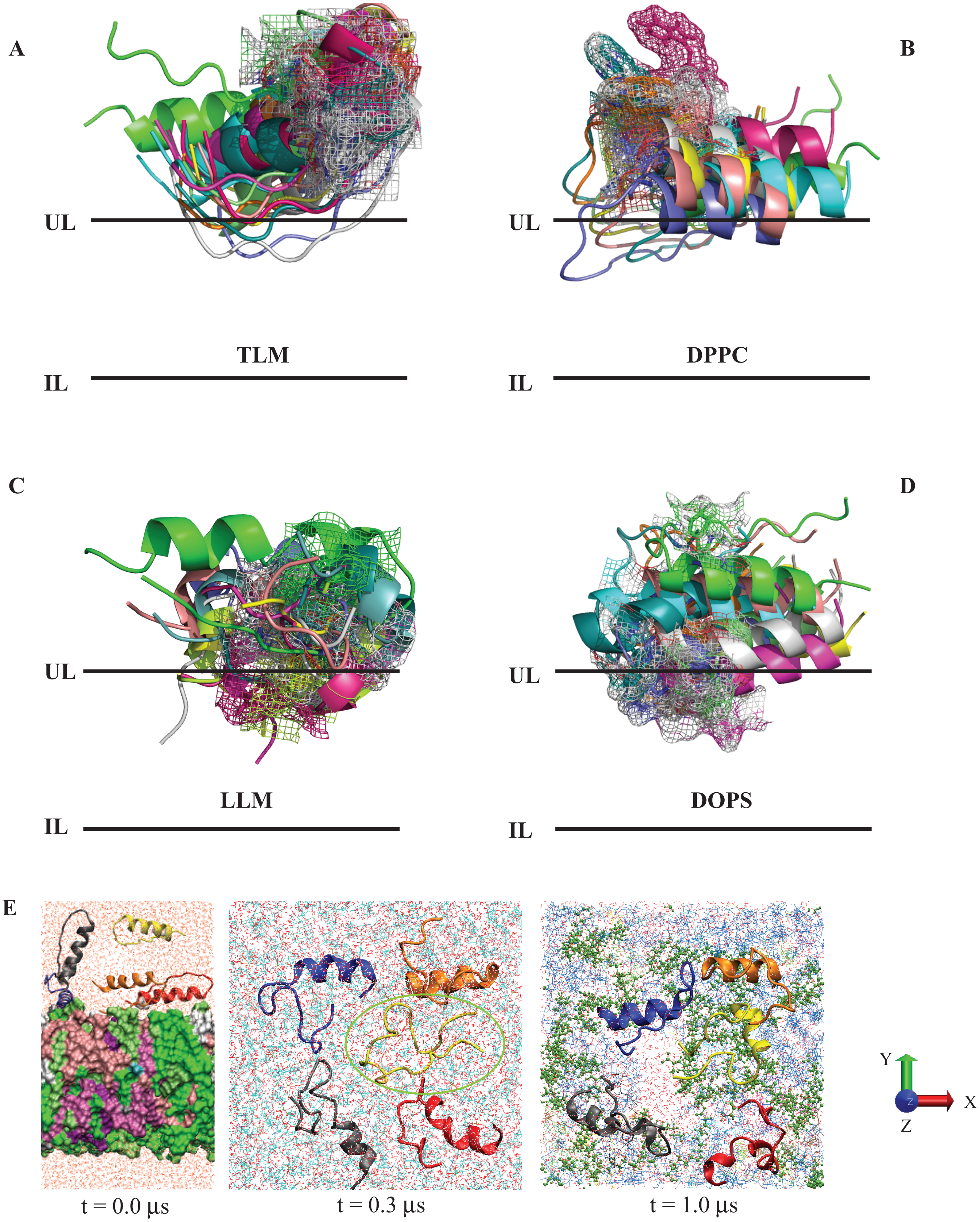


Fig. S3. Illustration of tryptophan (W14) position in different membrane system during MD simulation. A total of 1 0 snapshots retrieved at equal interval of time from the MD trajectory is superimposed and shown as a cartoon in PyMOL in (A) TLM; (B) DPPC; (C) LLM; and (D) DOPS systems. The W14 position is shown in a mesh and the outer and inner leaflet surface of bilayers are presented in solid horizontal lines for visualization transparency. (E) Structural rearrangement of five randomly distributed BMAP-28 molecules during 1 µs MD simulation in LLM system. The BMAP-28 monomers are represented by different colors and lipids as surface/lines. The circle shows the structural transition in aqueous phase, and the anionic lipid patches surrounding the peptide molecules are shown in green.

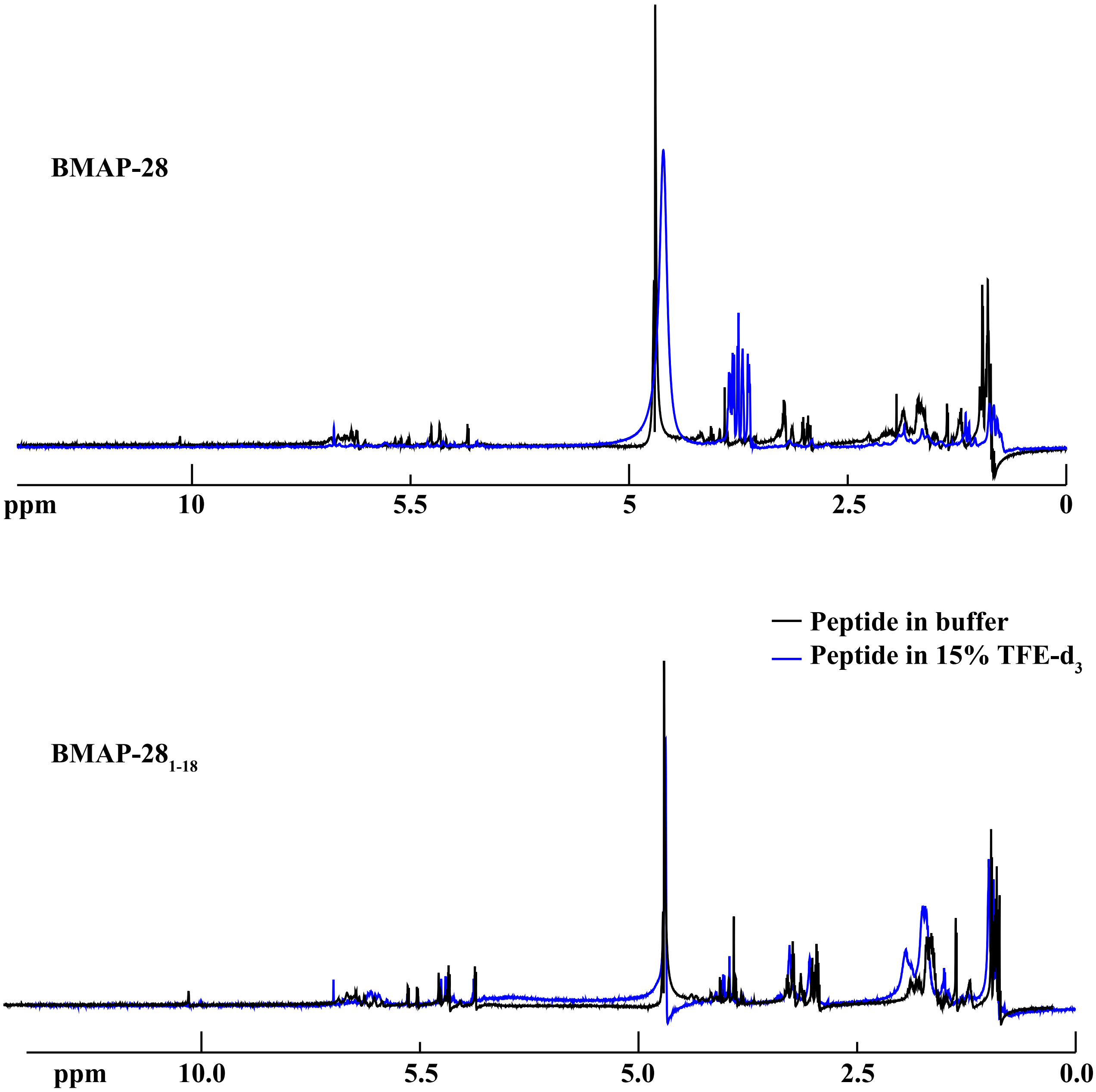


Fig. S4. 1D proton NMR spectra of 1.5 mM BMAP-28 and BMAP-$28_{1-18}$ in 25 mM sodium phosphate buffer containing 100 mM NaCl (pH=6.4) recorded at 700 MHz and 37° C. The NMR spectrum in buffer+D2O (90% + 10%) and buffer+D2O+TFE-d3 (75% + 10% + 15%) are shown in black and blue, respectively.

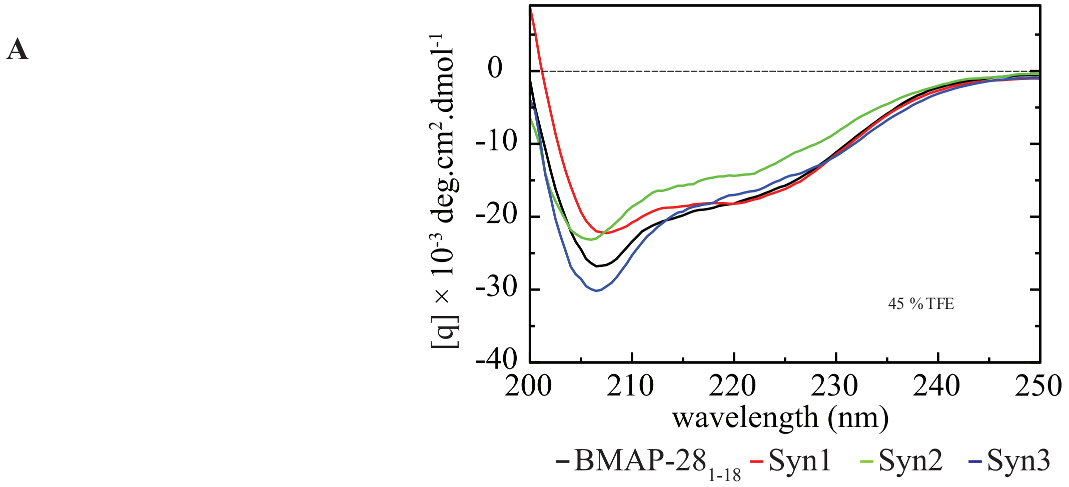


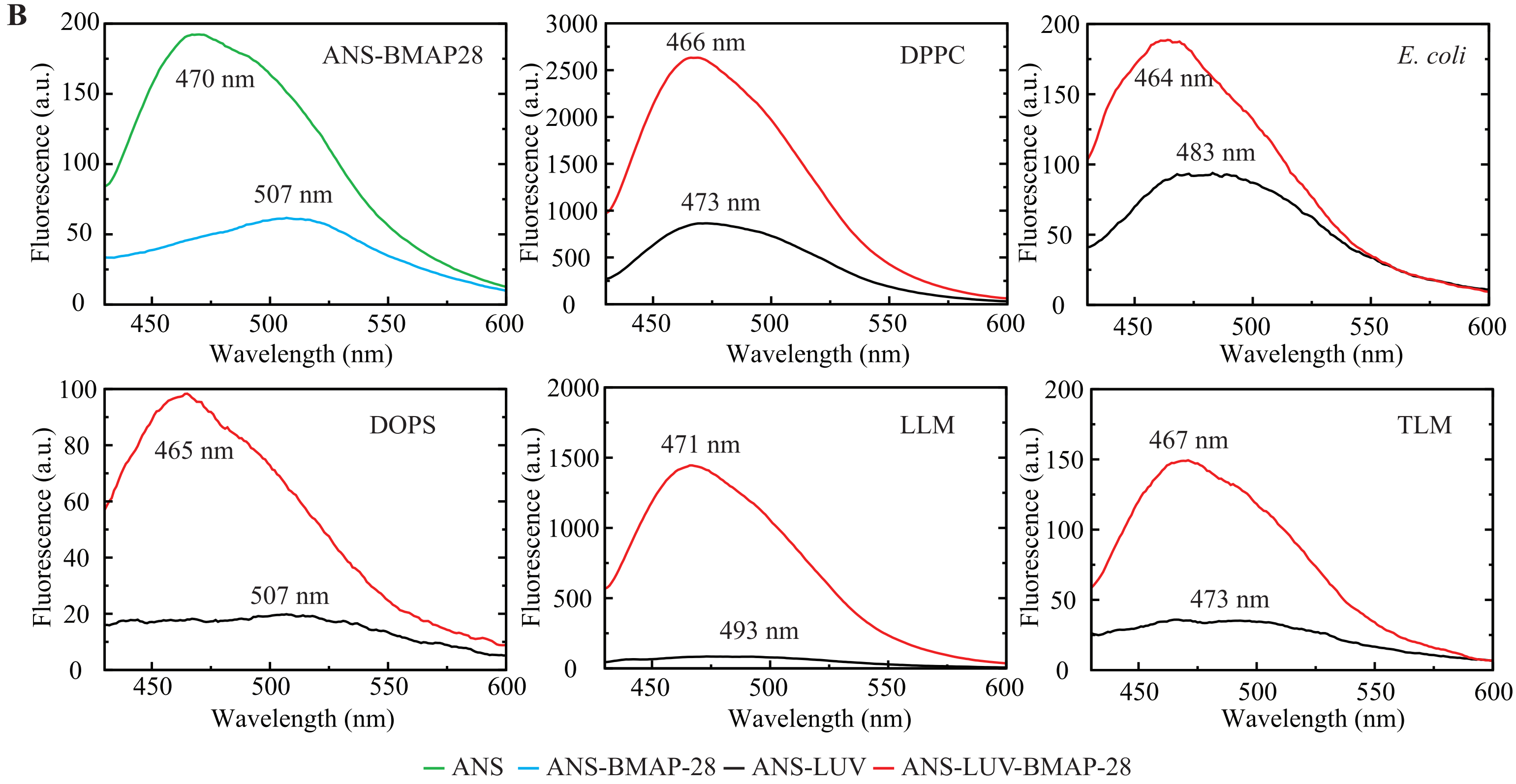


Fig. S5. (A) Circular dichroism spectra of BMAP-28 derivatives in 45% TFE solution mixture. (B) The fluorescence spectra of ANS binding assay at 2 μM in the presence and absence of BMAP-28 (2.5 μM) and LUVs. The horizontal and vertical axes represent the emission spectral wavelength and fluorescence intensity, respectively. (C) Determination of membrane mediated self-association and unfolding of BMAP-28$_{1-18}$ through ANS fluorescence spectroscopy. The fluorescence spectra of for ANS and ANS-peptide, ANS-liposome and ANS-peptide-liposome are shown in blue, green, black and red, respectively. The ANS-LUV and ANS-LUV- BMAP-28$_{1-18}$ spectra are shown in green and cyan, respectively. The spectra maximum are labeled in each membrane system.

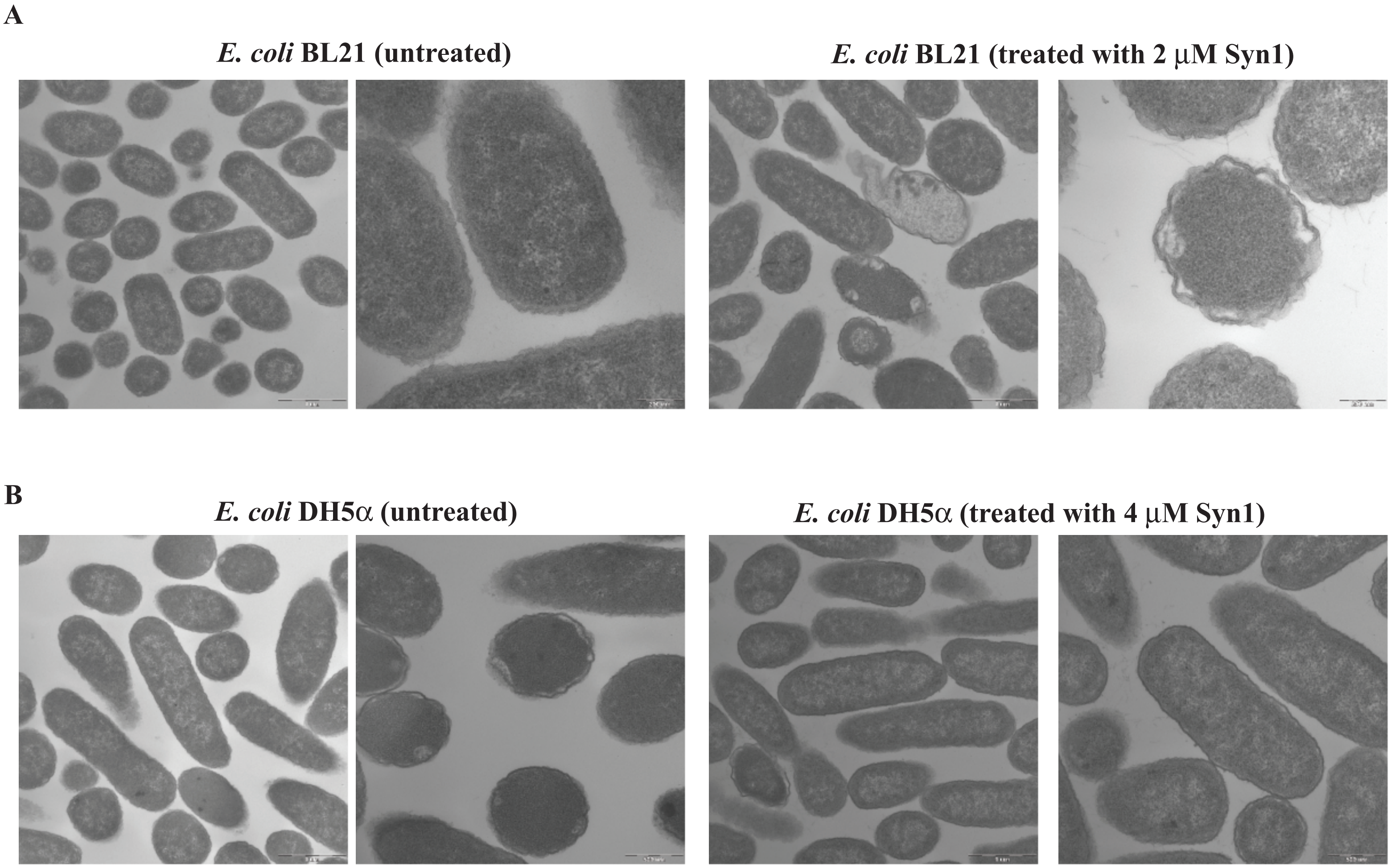


Fig. S6. Micrographs of transmission electron microscopy in *E.coli* BL21 cells treated with Syn1 at 2 μM (A); and DH5α cells at 4 μM (B). Two different illustrations of peptide untreated and treated cells are shown in the left and right column, respectively.

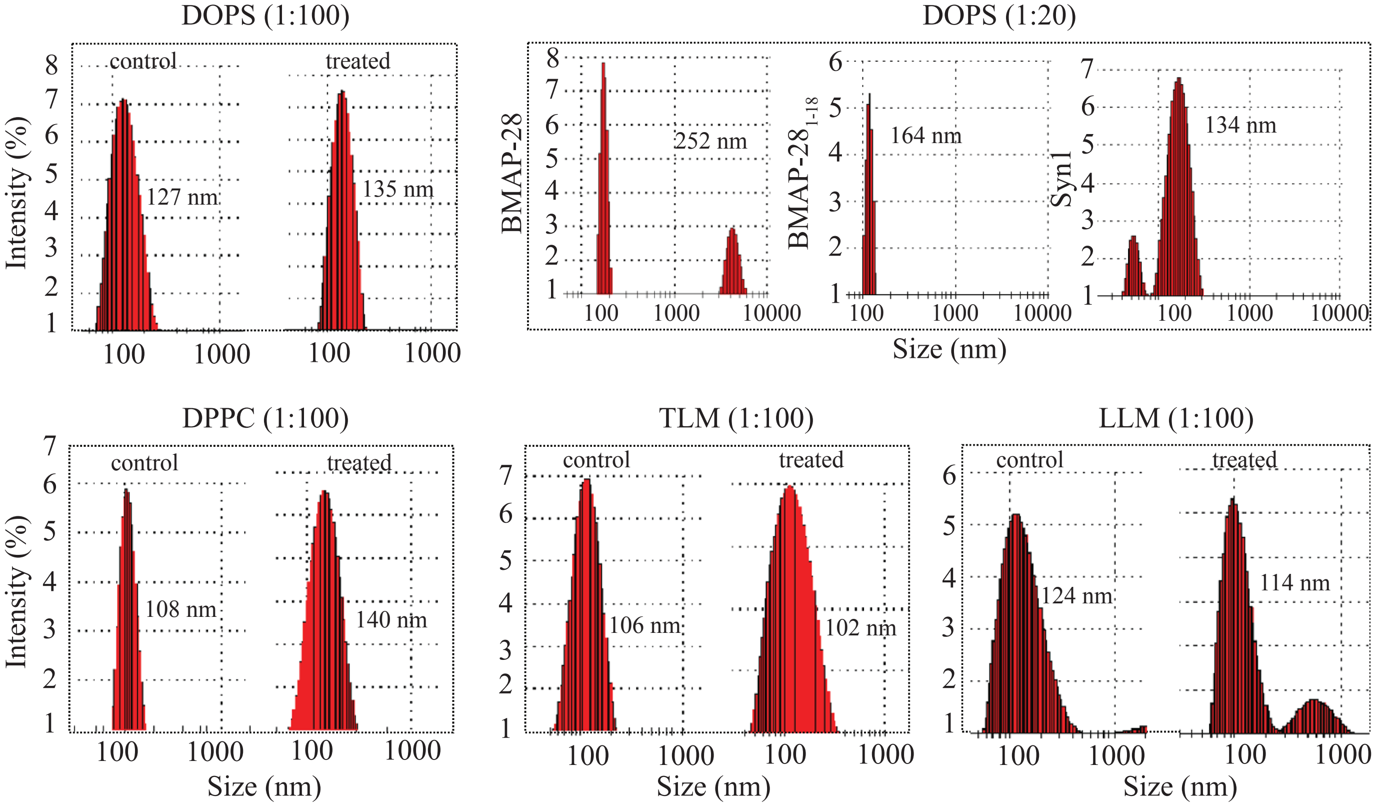


Fig. S7. Size distribution of LUVs studied by dynamic light scattering. The left and right panel shows the average size of LUVs with (treated) and without (control) peptide treatment. The DOPS, DPPC, TLM and LLM were treated with BMAP-28 at P/L= 1:100. The only DOPS LUVs are also treated with BMAP-28 analogues at a higher peptide concentration (P/L=1:20). The average sizes of the LUVs are shown inside the boxes.

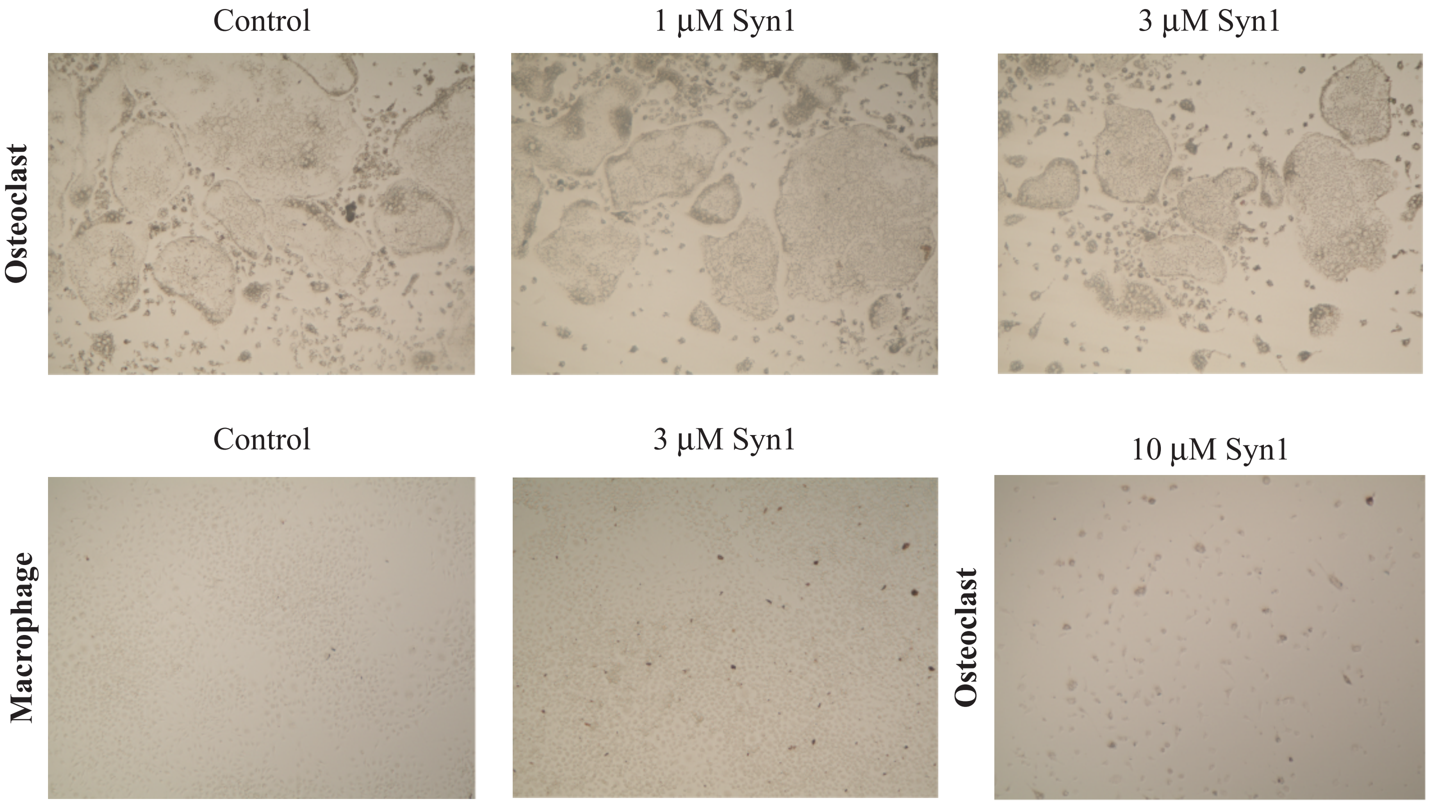


Fig. S8. Effect of Syn1 on osteoclasts and macrophages. The treated peptide concentrations are shown in the horizontal axis. The effect of target peptide on osteoclast and macrophage cells are observed on day 4. The inhibition of osteoclasts at 3 and 10 μM peptide concentration are shown in the right panel.
